# Vertical distribution and metabolic diversity of autotrophic microbes in the deep sediment of the challenger deep

**DOI:** 10.1186/s40793-026-00908-5

**Published:** 2026-05-12

**Authors:** Jiahua Zhou, Haojin Cheng, Yulin Zhang, Tianhang Liu, Xing Chen, David J. Lea-Smith, Jonathan D. Todd, Jiwen Liu, Xinxin He, Ronghua Liu, Xiao-Hua Zhang

**Affiliations:** 1 Frontiers Science Center for Deep Ocean Multispheres and Earth System, College of Marine Life Sciences, Ocean University of China, Qingdao, 266003 China; 2 Laboratory for Marine Ecology and Environmental Science, Laoshan Laboratory, Qingdao, 266237 China; 3 Institute of Evolution & Marine Biodiversity, Ocean University of China, Qingdao, 266003 China; 4 School of Systems Science, Beijing Normal University, Beijing, 100875 China; 5 School of Biological Sciences, University of East Anglia, Norwich, NR4 7TJ UK; 6 Quadram Institute Bioscience, Norwich Research Park, Norwich, NR4 7UQ UK

**Keywords:** Carbon fixation, Autotrophic microorganisms, Hydrogen, Redox conditions, Challenger deep

## Abstract

**Background:**

Carbon fixation in marine ecosystems is a vital process that contributes to climate regulation, with ocean sediments playing a critical role in carbon sequestration. This process is driven by chemolithoautotrophy in marine sediments, fueled by reduced compounds, such as those containing nitrogen and sulfur. However, the vertical distribution of microbial autotrophs and their energy coupling systems remain poorly understood in many sediments. In this study, we investigated a 750 cm sediment core from the Challenger Deep, the deepest point on Earth, which harbors abundant and diverse microbes under extreme conditions.

**Results:**

To explore the autotrophic characteristics across redox conditions in this core, we characterized the microbial community, metagenome, and metagenome-assembled genomes (MAGs), and their potential for carbon fixation processes and associated energy metabolism. The Wood-Ljungdahl (WL) pathway, primarily driven by *Planctomycetota* and *Aerophobota*, and the reverse oxidative TCA (roTCA) cycle, primarily driven by *Bacteroidota* and *Gemmatimonadota*, were the dominant predicted carbon fixation pathways, with hydrogen as the primary energy source, coupled to nitrogen and sulfur metabolism. Notably, the 3-hydroxypropionate/4-hydroxybutyrate (3HP/4HB) cycle, mediated by *Nitrososphaeria*, showed the highest abundance in the oxidized environment (15–27 cm below the seafloor), where ammonia oxidation likely served as the primary energy source. *Gammaproteobacteria* were predicted to utilise sulfur oxidation, whereas *Alphaproteobacteria* and *Chloroflexota* used hydrogen to drive the Calvin-Benson-Bassham (CBB), reductive glycine pathway (rGly) in *Alphaproteobacteria* and the dicarboxylate/4-hydroxybutyrate cycle (DC/4HB) in *Chloroflexota*, respectively. The abundance of carbon fixation, and nitrogen, sulfur and hydrogen cycling functional genes were significantly correlated with environmental factors (NH_4_^+^ and SiO_3_^2−^) based on Pearson’s correlation analysis.

**Conclusion:**

This study reveals the vertical distribution of microbial carbon fixation potential and diversity in sediments driven by redox conditions, highlights the crucial role of hydrogen as an energy source, and provides new insights for optimizing global deep-sea carbon cycle models. Collectively, these findings extend the redox tower theory by revealing a hadal-sediment specific distribution of autotrophic genes, characterized by persistent enrichment of energetically efficient pathways and dominant hydrogen-based energy coupling across deep sediment layers.

**Supplementary Information:**

The online version contains supplementary material available at 10.1186/s40793-026-00908-5.

## Background

Marine ecosystems capture atmospheric CO_2_ and store it in oceanic reservoirs through biological and physicochemical interactions [1]. Until recent decades, most studies on carbon capture focused on the marine epipelagic zone [2], but it is increasingly recognized that deep ocean microbes also drive carbon fixation [3–5], methane production and oxidation [6], and carbon degradation [7]. In particular, carbon fixation by chemolithoautotrophic microorganisms in marine sediments, of which ~ 85% are under 300 m [8], is fundamental to the dynamics of the global carbon cycle and climate regulation [9, 10].

From a bioenergetic and ecological perspective, microbial metabolic strategies are expected to be structured by trade-offs between energy yield, substrate availability, and environmental constraints. For example, eight pathways of autotrophic carbon fixation have been found [11–13]: the Calvin-Benson-Bassham (CBB) cycle, the reductive Acetyl-CoA or Wood-Ljungdahl (WL) pathway, the reductive tricarboxylic acid (rTCA) cycle, the 3-hydroxypropionate (3HP) cycle, the 3-hydroxypropionate/4-hydroxybutyrate (3HP/4HB) cycle, the dicarboxylate/4-hydroxybutyrate (DC/4HB) cycle, the reductive glycine (rGly) pathway, and the reversed oxidative TCA (roTCA) cycle. These pathways differ substantially in their energy efficiency, oxygen sensitivity, and cofactor requirements [14] and are increasingly recognized as important for primary production in aphotic marine environments [15–18]. For example, the CBB and rTCA cycles dominate carbon fixation in deep-sea vents [19, 20], the 3HP/4HB cycle, predominantly mediated by *Nitrososphaeria*, is likely significant in the world’s deepest blue hole [21], anaerobic methanotrophs primarily utilize the WL pathway in methane seeps in the Black Sea, Hydrate Ridge and Gullfaks [22, 23], and *Proteobacteria*, *Chloroflexota* and *Planctomycetes* couple sulfur oxidation with the rTCA and WL pathways in cold seeps of the South China Sea and trench sediments [24]. Carbon fixation, driven by oxidation of reduced inorganic compounds such as ammonia, hydrogen and sulfur, represents significant pathways of marine primary production in both photic and aphotic zones [3, 25] with variation in environmental constraints, notably redox conditions and energy availability, exerting strong selective pressure on pathway distribution [14].

Deep-sea sediments receive organic matter from the photic zone [26, 27], which is typically elevated in trench systems, where deposition and burial processes shape microbial community structure [28, 29]. Notably, hadal trench sediments have been reported to host locally elevated microbial abundances and higher metabolism compared to adjacent abyssal plains, reflecting enhanced organic matter deposition [28, 30, 31]. Hadal trenches like the Japan Trench show higher total organic carbon (TOC; 1.63–3.28 wt%) and total nitrogen (TN; 0.22–0.42 wt%) than abyssal plains due to high sedimentation, terrigenous input, marine productivity, and trench-specific processes (e.g., lateral transport, rapid burial) that accumulate organic matter [32, 33].

The Mariana Trench, situated in the western Pacific Ocean, is recognized as the deepest known oceanic trench, reaching a maximum depth of 10,983 ± 6 m at the Challenger Deep [34, 35]. The majority of the trench floor lies below 8,000 m and is characterized by extreme environmental conditions, including high hydrostatic pressure, low temperature, limited oxygen availability and complete darkness. These unique environmental characteristics select for highly specialized microbial communities with distinct physiological and metabolic adaptations [36]. Metagenomic analyses of microbial communities from axial and slope sediments uncovered a wealth of previously uncharacterized microbial lineages, underscoring the distinct and diverse microbial ecosystems that inhabit ultra-deep environments [37–39]. Similarly, studies of hadal sediments in the Mariana Trench emphasized the important role of microbial communities in sulfur cycling [36, 40]. While previous investigations have shown that vertical shifts in benthic community structure and abundance correlate with organic carbon (OC) accumulation and variations in redox conditions [33], autotrophic carbon-fixing populations in the trench sediments remain poorly characterized, leaving unresolved how redox stratification and electron donor-acceptor dynamics interact to govern the vertical organization of carbon fixation pathways in hadal sediments.

It is unclear how carbon fixation pathways are vertically organized across redox gradients in hadal sediments, which energy conservation mechanisms support autotrophy under steep redox gradients and constrained electron acceptor availability, and whether hydrogen functions as a primary driver of anaerobic carbon fixation in the deepest sediment layers. To address this knowledge gap, we analyzed the potential for microbial carbon cycling in a 7.5 m sediment core retrieved from the Challenger Deep in the Mariana Trench. Metagenomic sequencing and metagenome-assembled genome (MAG) analysis were used to resolve the vertical stratification of microbial carbon fixation pathways and their coupled energy conservation mechanisms across redox gradients. We demonstrate that redox stratification strongly structures the vertical distribution of microbial carbon fixation pathways in Mariana Trench sediments. Energetically efficient pathways, particularly the WL and roTCA cycles, dominate carbon fixation in deep sediment layers. We further predict that hydrogen metabolism is closely linked to these carbon fixation strategies and provides a key energy source supporting metabolically flexible mixotrophic lifestyles in hadal microbial communities.

## Methods

### Sample and environmental parameter collection

The continuous sediment core (752 cm in length) was derived from the Challenger Deep in the Mariana Trench (MT20-750, 11° 19.904ʹ N, 142° 12.083ʹ E) aboard the R/V *Dong Fang Hong 3* in July 2020 [36]. The sediment core was sectioned and subsampled at predefined depth intervals using a stainless-steel cutter and sterilized spatulas [36]. To avoid contamination, the outer 1 cm layer of deep sediment was removed and the uncontaminated central portion of the core was collected into sterilized plastic tubes and stored at -80 ℃ until DNA extraction. The internal pore water was extracted in accordance with previous studies [32, 33] for subsequent environmental parameter measurements. The porewater nutrients (NO₃⁻, NO₂⁻, NH₄⁺, PO₄³⁻, SiO₃²⁻) were determined using a QuAAtro continuous-flow analyzer (SEAL, Germany). Porewater sulfate was analyzed using ion chromatography (CIC-D100, Shine, China) on 1:80 diluted aliquots in Milli-Q water. Sediment TOC and TN were analyzed using a FLASH 2000 elemental analyzer (Thermo Fisher Scientific) following standard procedures [41]. The analytical precision (standard deviation, *n* = 6) was ± 0.02 wt% for TOC and ± 0.01 wt% for TN. Sediment grain size distributions were determined using a Mastersize 3000 laser particle size analyzer. In total, porewater nutrients, sediment TOC, TN, and grain size data from 37 discrete depth intervals throughout the entire core were used for correlation analyses.

### Metagenomic assembly and MAG generation

As previously outlined, sample DNA was extracted from 10–12 g of sediment from 22 representative depth intervals of the sediment core using a phenol-chloroform method [36]. DNA libraries were prepared without amplification by BGI Tech Solutions (Beijing Liuhe) Co., Ltd. Briefly, 1 µg of qualified DNA was fragmented using a Covaris ultrasonic instrument, and fragments of 200–400 bp were selected using magnetic beads. The fragments were end-repaired, A-tailed, and ligated with sequencing adapters. Adapter-ligated products were amplified by PCR and purified using magnetic beads. The purified PCR products were denatured into single strands and circularized to generate single-stranded circular DNA. Uncircularized linear DNA molecules were digested, yielding the final libraries. The libraries were quantified before sequencing. Metagenomic sequencing was then performed on an Illumina HiSeq X-Ten platform, generating 2 × 150-bp paired-end reads, which are also available from NCBI (PRJNA957232 and PRJNA957236).

Consistent with our previous study, metagenomic assembly was performed following the approach described in Liu et al. [33]. Briefly, raw sequencing reads were processed using the metaWRAP-Read_qc module to eliminate adapters and low-quality sequences. Adapters and low-quality bases were removed using TrimmomaticPE (v0.39) in paired-end mode. Bases with quality scores below 3 at the 5’ or 3’ ends were trimmed, and a sliding window approach was applied to remove regions with an average quality score below 15. Reads shorter than 75 bp after trimming were discarded. No host genome removal was performed, as samples with environmental and minimal host contamination was expected. Cleaned reads were quality-assessed using FastQC and were subsequently assembled into contigs using MetaSPAdes v3.15.2. Contigs from each sample underwent binning with three metagenomic binning tools (MaxBin2 v2.2.4, metaBAT2 v2.12.1, and CONCOCT v0.4.0). Bins were integrated through the Bin_refinement module to produce metagenome-assembled genomes (MAGs). The quality of the MAGs was evaluated using CheckM v1.0.7, and MAGs with > 50% completeness and < 10% contamination were retained for further analysis.

The relative abundance of genes and MAGs was assessed as previously described in Cheng et al. [36]. Briefly, RPKM (reads per kilobase per million mapped reads) values were calculated by mapping reads with BWA-MEM and processing with samtools. Reads with coverage of < 80% were filtered using CoverM filter v0.4.0 (Ben Woodcroft, CMR, QUT, UK).

### Gene and functional annotation

The metagenomic sequences and individual MAG were analyzed for gene prediction using prodigal version 2.6.3 with default settings [42]. For carbon fixation functional annotation, protein sequences were annotated using DIAMOND software (v0.9.9) with the BLASTp algorithm using an E-value cutoff of 1e-10 against the marker genes obtained from the KEGG protein database [43]. The accuracy of annotation was enhanced through three complementary approaches. First, alignment with the KEGG database using the GhostKOALA module. Second, comparison with the HMM (E-value < 1e-10) database of key enzyme genes from eight known carbon fixation pathways. These HMM profiles were compiled from KEGG Orthologs definitions and published literature [12, 21, 25] and are listed in Supplementary Table S13. Third, identification of proteins with > 40% identity using BLASTp (E-value < 1e-10) against genes involved in carbon fixation pathways and other energy metabolism pathways. The key enzymes used for BLASTp-based validation of carbon fixation and energy metabolism pathways are summarized in Supplementary Table S13.

Nitrogen- and sulfur-related genes were identified via KEGG annotation, while hydrogenases were annotated using BLASTp against reference protein databases (10.26180/c.5230745). Alignments were then filtered using minimum cutoffs of sequence identity of 60% for FeFe hydrogenases and NiFe Group 4 hydrogenases, and 50% for Fe hydrogenases and other NiFe hydrogenase groups.

To further confirm pathway completeness, we rigorously assessed the completeness of specific metabolic pathways in individual MAGs by reconstructing entire pathways using KEGG-Decoder (https://github.com/bjtully/BioData/tree/master/KEGGDecoder, Los Angeles, CA, USA). Here, pathway completeness was evaluated using different strategies depending on the characteristics of each carbon fixation pathway. For the CBB, rTCA, 3HP, and 3HP/4HB pathways, completeness was calculated using KEGG-Decoder, whereas for the DC/4HB, rGly, and roTCA pathways, completeness was assessed based on the presence of all known essential functional genes involved in carbon fixation. For the WL pathway, the completeness of both the methyl and carbonyl branches was explicitly evaluated by examining the presence of branch-specific key functional genes. Since not all genomes containing carbon fixation pathways are strictly autotrophic, the core metabolic pathways in the screened carbon fixation MAGs were also analyzed. The bubble diagram and heatmap were generated with Python 3.11. The relative abundance of specific pathways was evaluated and compared using DiTing (https://github.com/xuechunxu/DiTing, Version 0.5) with an unbiased specificity formula [44]. The taxonomy of the 1766 MAGs (bins) was classified by GTDB-Tk v220 [45].

### Analysis of the energy contribution to the carbon fixation pathways

The energy contribution of each electron donor was model-estimated based on predicted metabolic potential inferred from sequence-derived functional annotations, gene coverage, and relative abundance across redox conditions, using the Gibbs free energy change (ΔG) associated with each corresponding donor-acceptor metabolic reaction under environmentally relevant conditions [46]. Specifically, the energy contribution ratio of each electron donor was calculated following the framework described in Zhou et al. [46] (https://github.com/AnantharamanLab/Hydrothermal_plume_omics_Zhou_et_al._2021), in which predicted sequence-based energy metabolisms are weighted by reaction energetics and normalized across all considered donors. To simplify the redox conditions in the core sediment, we only considered two major electron acceptors (O_2_ and NO_3_^−^) and used their relative abundance ratio to infer the contribution of electron donors under different levels of oxidative conditions.

### Extraction of shared interactions and determination of potential exchangeable metabolites

Co-occurrence features of paired MAGs were detected by co-occurrence networks. Metabolic complementation networks (https://inap.denglab.org.cn/) [47] were constructed using the PhyloMint algorithm to assess the potential for metabolic co-operation between MAGs involved in carbon fixation and from other MAGs lacking carbon-fixation potential. In the PhyloMint algorithm, seed metabolites (representing exogenous substrates) were defined based on strongly connected components, and the potential for metabolite utilization between two MAGs was assessed by comparing the degree of overlap between the set of seed metabolites of one MAG and the set of unseeded metabolites of another MAG. Metabolite profiles were derived from genome-scale metabolic models generated by CarveMe using the BiGG model database (version 1.6). Metabolites were classified into nine superclasses based on manually revised classification rules from the HMDB (version 5.0), including coenzyme A derivatives, amino acids/peptides and analogues, carbohydrates and conjugates, inorganics, and organic compounds with specific atoms or structures.

### Correlation analysis

We quantified the correlation between physicochemical properties (listed in Table S5) and carbon, nitrogen and sulfur functional genes, which were obtained by annotation with the GhostKOALA module. To ensure comparability across physicochemical properties and carbon, nitrogen and sulfur functional genes, all variables were first standardized using z-score normalization (subtracting the mean and dividing by the standard deviation). Pearson’s correlation was then used to test the association between each environmental factor and the abundance of each functional gene. Statistical significance was set at *p* < 0.05, false discovery rate (FDR) corrected [48]. To account for potential confounding effects among environmental variables, sampling depth (calculated as the mean depth of each sampling site) and all environmental factors except the current one to be tested were used as covariates regressed out by a generalized linear model (GLM) [49] from both environmental factors and functional gene abundance.

### Other carbon metabolic reconstruction

The dbCAN web server, which integrates HMMER, DIAMOND, and Hotpep tools [50], was used to identify carbohydrate-active enzyme (CAZy) genes. Heatmap analysis was performed using all MAGs to investigate KEGG genes associated with eight carbon fixation pathways. In addition, the relative abundance of CAZy genes across all metagenomic samples was visualized using heatmaps, and the number of CAZy genes within individual MAGs was quantified.

### Phylogenetic analyses of the reconstructed genomes and key metabolic genes

Maximum likelihood trees were constructed by screening marker genes from KEGG annotation to infer phylogenetic relationships of different functional proteins among 539 bacterial MAGs or 5 archaeal MAGs. Bac120 or arc122 proteins were predicted using the GTDB-Tk identify module. Concatenated multiple sequence alignment was performed with the GTDB-Tk align module. The phylogenomic trees of MAGs were inferred with IQ-TREE version 1.6.1 under the LG + R10 and LG + R4 models. The phylogenetic trees of related key genes were constructed and performed by MAFFT [51] and IQ-TREE [52] with default options. The phylogenetic trees were annotated using the Interactive Tree of Life (iTOL) webtool for better visualization [53].

## Results

### Carbon fixation pathway abundance in the deep sediment sample

**Fig. 1:**
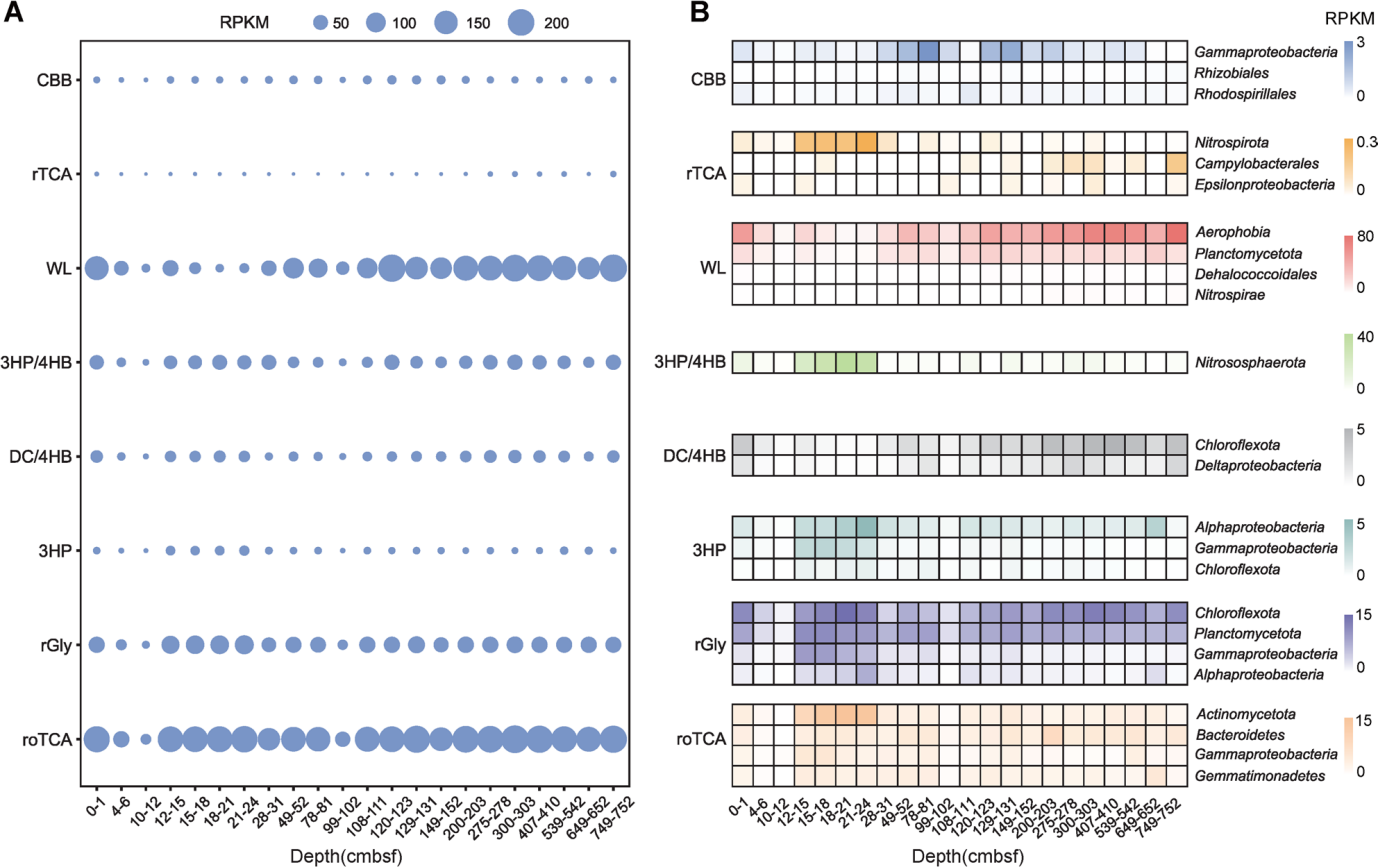
Vertical distribution of microbial carbon fixation pathways and dominant taxa in Mariana Trench deep sediment.** A** Vertical distribution of microbial carbon fixation pathways in Mariana Trench deep sediment. Bubble plots show the depth-resolved relative abundance of the pathways. Pathway abundance was calculated as the average abundance (RPKM) of all marker genes.** B** Dominant microbial taxa contributing to different carbon fixation pathways. Color intensity represents the relative abundance (RPKM) of marker genes assigned to each taxon. Taxonomic labels represent mixed taxonomic ranks (phylum, class, or order). Full details of marker gene sets, KEGG Orthology identifiers or HMM accessions, detection thresholds, and pathway definition criteria are provided in Table S13. CBB, Calvin cycle; WL, Wood-Ljungdahl pathway; rTCA, reductive tricarboxylic acid cycle; 3HP, 3-hydroxypropionate cycle; 3HP/4HB, 3-hydroxypropionate/4-hydroxybutyrate cycle; DC/4HB, Dicarboxylate/4-hydroxybutyrate cycle; rGly, Reductive glycine pathway; roTCA, Reverse oxidative TCA cycle

To explore the microbial metabolic strategy of inorganic carbon acquisition in deep-sea sediments, we determined the relative abundance of marker genes (Fig. 1A). From least to most abundant across the entire core were the rTCA (0.01–0.66%), CBB (0.7–3.61%), 3HP (0.73–4.54%), DC/4HB (2.86–7.33%), 3HP/4HB (5.46–15.96%), rGly (10.35–22.77%), WL (2.67–38.48%) and roTCA (33.81–49.35%) pathways, with variation in abundance within regions. Notably, the potential for all pathways showed an abrupt abundance decline at 10–12 cmbsf and 99–102 cmbsf, suggesting depth-related shifts in carbon fixation. These changes were not directly related to differences in the measured geochemical parameters (Table S5), and were possibly due to the complex environmental controls on carbon fixation in sediments. Also, the relative abundance of the 3HP/4HB pathway genes increased from 5.46–9.53% to 10.88–15.96% under oxidative conditions as described previously [33], indicating potential sensitivity of this pathway to local redox conditions.

### Distributional patterns of potential bacterial autotrophs in the deep sediment

**Fig. 2:**
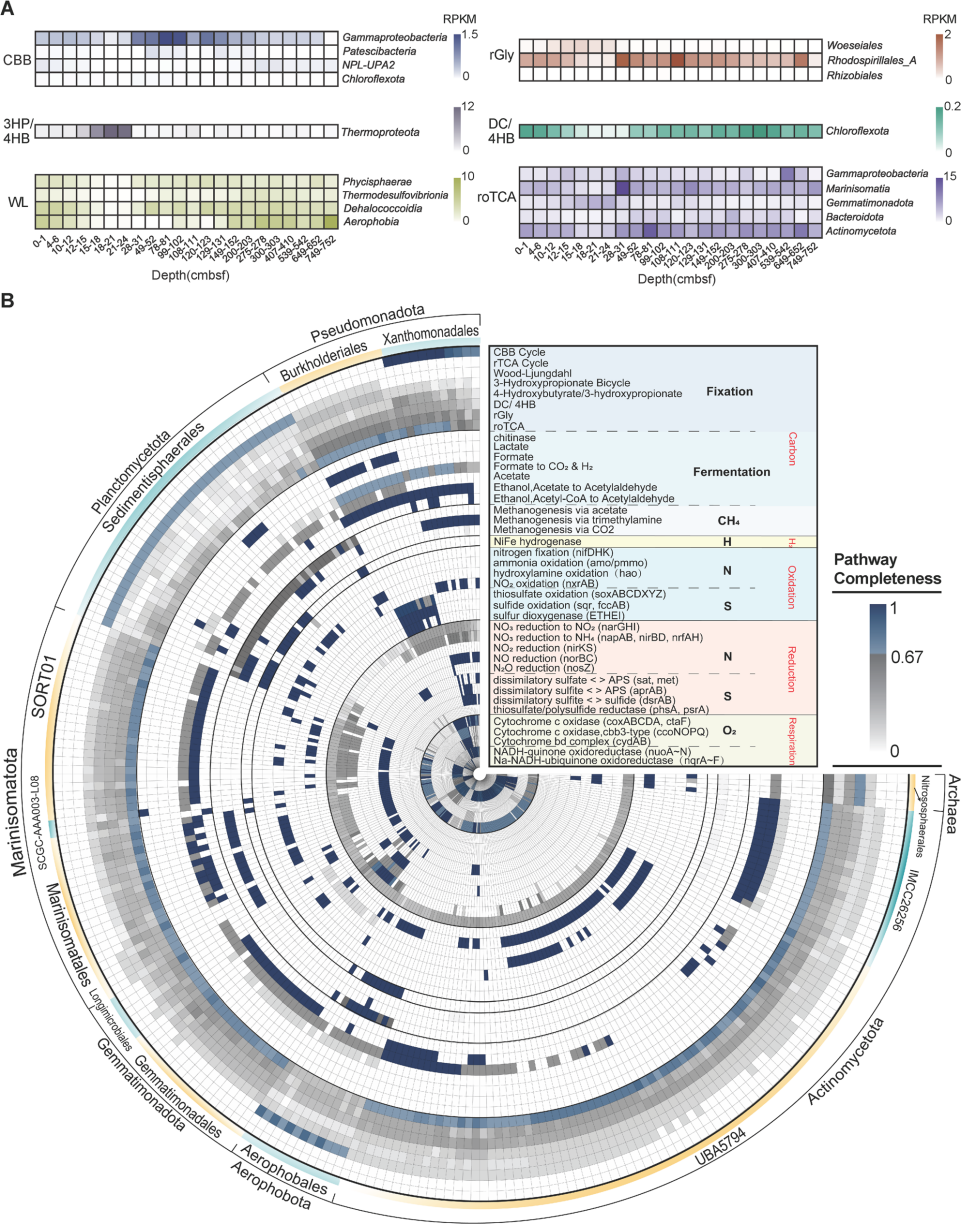
The coupled redox metabolic features of autotrophic MAGs in the deep sediment. **A** Vertical distribution patterns of major microorganisms involved in the carbon fixation pathway. The data are shown as heatmaps, where color intensity represents normalized gene abundance (RPKM). Taxonomic labels are shown at mixed ranks (phylum, class, or order). **B** Energetic metabolism of autotrophic MAGs in deep sediment. MAGs encoding carbon fixation pathways with a completeness > 67% are displayed. Heatmap showed the completeness of each pathway. CBB, Calvin cycle; WL, Wood-Ljungdahl pathway; rTCA, reductive tricarboxylic acid cycle; 3HP, 3-hydroxypropionate cycle; 3HP/4HB, 3-hydroxypropionate/4-hydroxybutyrate cycle; DC/4HB, Dicarboxylate/4-hydroxybutyrate cycle; rGly, Reductive glycine pathway; roTCA, Reverse oxidative TCA cycle

To explore the distribution of bacterial autotrophs involved in carbon fixation in hadal sediments, we analyzed bacterial community composition (Fig. 2, Table S1). A total of 544 MAGs (from 1766 MAGs) were identified as harboring potential carbon fixation pathways, of which 502 (92.3%) had incomplete pathways (0.67-1.0) and 42 (7.7%) had complete pathways. From these 42 MAGs, 12 harbored genes putatively encoding both RuBisCO subunits of the CBB cycle, with most in *Xanthomonas* gammaproteobacteria and mainly distributed at depths of 78–102 cmbsf. Since members of this genus are typically associated with terrestrial plant environments, their occurrence in hadal sediments may reflect long-range transport via terrestrial runoff and subsequent deposition through particulate organic matter sinking.

Genes potentially encoding all key enzymes of the CBB cycle were identified in 33 MAGs, specifically *Poribacteria*, *Pseudomonadota* and *Chloroflexota*. Multiple MAGs with relatively high abundance were identified at 15–27 cmbsf, including four *Nitrososphaerales* containing genes potentially encoding 3HP/4HB pathway enzymes and *Gemmatimonadota* with genes potentially encoding roTCA pathway enzymes (Figs. 1A, 2B). The co-occurrence of distinct autotrophic taxa and pathways at this depth coincided with redox transition zones in the sediment. MAGs containing genes potentially encoding rGly pathway enzymes were exclusive to *Pseudomonadota* and exhibited significantly lower abundance in oxidized environments at 15–27 and 749–752 cmbsf depths. This contrasting depth distribution suggests differential environmental preferences among carbon fixation strategies.

Genes potentially associated with the WL pathway were identified in 178 MAGs assigned to *Planctomycetes*, *Nitrospirota*, *Desulfobacterota*, *Aerophobota* and *Chloroflexota*. However, the presence of WL marker genes alone does not necessarily indicate a complete autotrophic pathway. Detailed analysis revealed that 130 MAGs encoded key enzymes of the WL carbonyl branch, including 30 containing putative genes specific to the H_4_F-dependent methyl branch (Table S10). MAGs with putative WL pathways from *Planctomycetes* were predominantly at 120–123 cmbsf, while those from *Aerophobota* were abundant in deeper sediment at 749–752 cmbsf. This depth-dependent distribution of WL-associated taxa may reflect stratification of anaerobic autotrophic niches within the sediment.

A total of 354 MAGs contained genes potentially encoding enzymes of roTCA pathways (completeness between 0.67 and 1). *Actinomycetota* was the dominant phylum, followed by *Pseudomonadota*, *Marinisomatota*, *Gemmatimonadota*, *Bacteroidota* and *Desulfobacterota*. The roTCA pathway in oxidative layers of the sediment (15–27 cmbsf) were predominantly in *Gemmatimonadales* MAGs, whereas those in *Marinisomatota* MAGs were predominantly in microoxic environments (28–31 cmbsf). *Burkholderiales* (*Pseudomonadota*) showed exceptionally high abundance at 539–542 cmbsf. The taxon-specific and depth-dependent distribution of the roTCA pathway suggests that this carbon fixation strategy is widely adaptable across sedimentary redox gradients.

### Energy coupling associated with carbon fixation

**Fig. 3:**
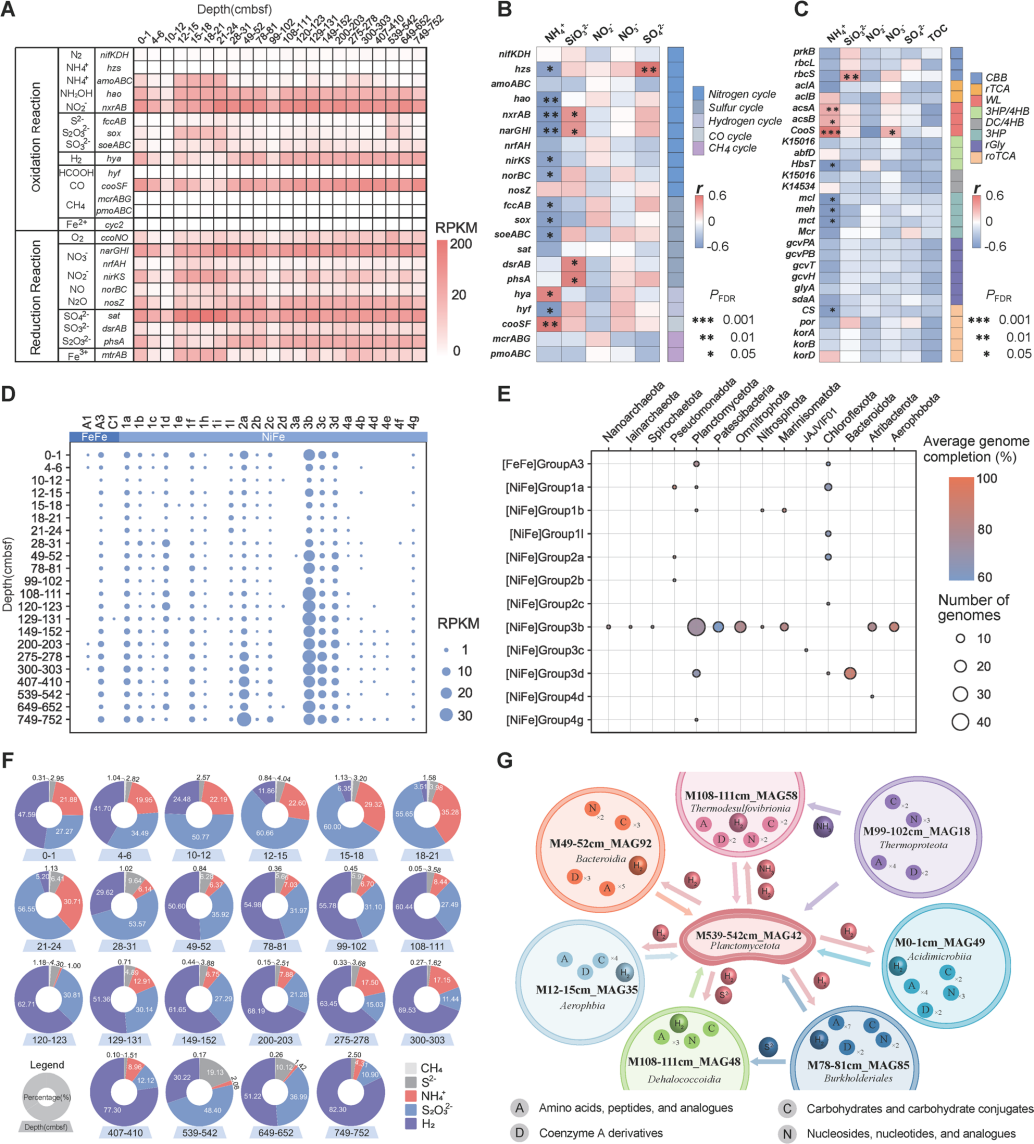
Electron donors, acceptors, and transferable metabolites in autotrophic carbon fixation of Mariana Trench deep sediment. **A** Marker gene abundance for electron donors and acceptors across depths. **B-C** Correlations between key N, S, and H₂ cycling genes (**B**), key carbon fixation functional genes (**C**) and environmental factors. Pearson’s correlation analyses were conducted between the abundance of each functional gene and each environmental factor. All environmental factors including depth except the current one to be tested were used as covariates regressed out by generalized linear model from both environmental factors and functional gene abundance. **D** Distribution of hydrogenases with depth in Mariana Trench deep sediment core. **E** Distribution of hydrogenases in carbon-fixing bacteria in the deep sediment core. The abundance of hydrogenases is summarized at phylum level, as the size of the circle corresponding to the number of MAGs harboring at least one given gene, and the color reflecting the pathway’s completeness in MAG. Marker genes that were not detected in any MAGs were omitted. **F** Energy contribution by various electron donors. The contribution is calculated based on reaction Gibbs free energy and metabolic potential predicted from metagenome. **G** Predicted metabolite transformation between autotrophic MAGs. Representative MAGs were selected and shown in this figure. The arrows indicate the predicted direction of metabolite transfer and each sphere represents a class of metabolite. *Planctomycetota* M539-542cm_MAG42 serves as the recipient of the metabolite NH_3_, S^2−^ and H_2_, which are represented by the spheres in the figure

Analysis of genes encoding enzymes likely involved in nitrogen and sulfur cycling, as well as metal reduction in deep sediment (Figs. 2B, 3A), revealed that energy conservation at 0–27 cmbsf probably occurs via multiple metabolic pathways. The abundance of these genes generally decreased from 0–12 cmbsf, with the lowest amounts at 10–12 cmbsf. Genes encoding enzymes likely involved in nitrite (*nirKS*), sulfate (*sat*), and metal (iron/manganese; *mtrAB*) reduction were ubiquitously distributed throughout the sediment. However, an increase in these genes and those likely encoding key enzymes for ammonia (*amoABC*), thiosulfate (*sox*), methane (*pmoABC*), NH_2_OH (*hao*), NO_2_^−^ (*nxrAB*), and CO (*cooSF*) oxidation, as well as NO_3_^−^ (*narGHI*), N_2_O (*nosZ*), SO_4_^2−^ (*sat*), and S_2_O_3_^2−^ (*phsA*) reduction, was observed at 15–27 cmbsf. The enrichment of these oxidation-reduction associated genes at intermediate depths coincided with zones of carbon fixation pathways, suggesting a potential association between electron-accepting and autotrophic processes.

### Microbial diversity of energetic metabolism associated with carbon fixation

To explore the energy sources for autotrophic microbes, nitrogen, sulfur, and hydrogen metabolism of predicted autotrophic MAGs was investigated (Fig. 2B). MAGs contained genes encoding putative enzymes of complete CBB cycles and those involved in nitric oxide reduction, dissimilatory sulfite and sulfide oxidation, acetaldehyde fermentation, methanogenesis, and aerobic respiration (Fig. 2B). MAGs of *Nitrososphaeria*, which fix carbon via the 3HP/4HB pathway, likely couple carbon fixation with both oxidative and reductive N/S metabolism, such as reductive sulfate metabolism (*sat*), NO reduction (*norBC*), sulfide (*sqr*) and ammonia oxidation (*amoABC*) (Fig. 2B).

MAGs of microbes with putative WL pathways, mostly *Planctomycetota*, showed possible distinct energy utilization strategies. *Pirellulales* MAGs contained genes encoding putative enzymes coupling sulfate reduction metabolism (*sat*), hydroxylamine oxidation, respiration, and fermentation linked to carbon fixation (Table S3). *Sedimentisphaerales*, an order within the phylum *Planctomycetota* (Fig. 2B, Fig. S3), may predominantly use hydroxylamine oxidation (*hao*) as an electron source for energy conservation (Fig. 2B). *Brocadiales* likely derived energy from hydroxylamine utilization (*hao*) and fermentation processes. None of the *Brocadiales* MAGs contained annotated genes for anaerobic ammonium oxidation (anammox). MAGs taxonomically classified as *Aerophobota* contained putative sulfide oxidation genes encoding fccAB-type sulfide dehydrogenases that may act as electron sinks facilitating redox balancing and contributing to energy conservation during sulfur transformation. The distribution of these types of energy metabolism among WL-associated taxa suggests that this pathway may be linked to a range of electron-accepting processes across reduced sediment horizons.

RoTCA-dependent autotrophic groups exhibited divergent potential energetic strategies at the phylum level. These MAGs contain genes suggesting that carbon fixation is coupled with different redox processes rather than relying on a single conserved metabolic strategy. Genes encoding putative thiosulfate oxidases (*sox*) and quinone oxidoreductases (*sqr*, *fccAB*) were detected within *Burkholderiales* MAGs (Fig. 2B), potentially serving as electron sinks mediating the roTCA pathway. *Marinisomatota* SORT01 contains genes potentially encoding key enzymes involved in sulfur and nitrogen transformation, including sulfite (*sat*), thiosulfate (*sox*) and hydroxylamine (*hao*) oxidation, and nitrate reduction (*narG*) (Fig. 2B; Table S3). In addition, genes encoding enzymes potentially involved in carbon fixation were detected (Fig. 2B, Table S13), indicating the potential for autotrophic carbon assimilation. The presence of fermentation genes suggests metabolic flexibility, allowing *Marinisomatota* to potentially switch between respiratory and fermentative energy strategies under varying environmental conditions. *Gemmatimonadales* may be capable of carbon fixation, potentially coupled with sulfur and nitrogen reduction processes (encoded by *sat*, *norBC*, *napAB*, *nrfAH*) that may function as electron sinks, whereas *Longimicrobiales* likely rely on fermentation and respiration. The co-occurrence of genes encoding putative enzymes of the roTCA cycle, sulfide oxidation and respiration via quinone oxidoreductases (*sqr*, *fccAB*) in *Dehalococcoidia* MAGs suggests that internal electron transfer may play a significant role in their carbon fixation. The *Actinomycetota* UBA5794 MAGs contained genes encoding potential nitrate reductase (*nxrAB*) and sulfur oxidase (*fccAB*) enzymes, while IMCC26256 may be capable of carbon fixation supported by fermentation and microaerobic respiration. Furthermore, *Acidobacteriota* UBA890 likely conducts NO_2_ oxidation and hydroxylamine oxidation, as it contains genes encoding potential nitrite reductase (*nxrAB*) and hydroxylamine oxidase (*hao*) enzymes, suggesting potential coupling between nitrogen transformation and autotrophic metabolism. Taken together, these results indicate that roTCA-associated lineages employ lineage-specific combinations of metabolic processes linked to carbon fixation, underscoring functional diversification among carbon-fixing taxa in Mariana Trench sediments.

### Coupling of hydrogenases to carbon fixation

Hydrogenases and other electron transfer agents (ETAs) were used as indicators of energy metabolism modality. A total of 26 genes (67%) were annotated as hydrogenases in the deep sediment, separated into [FeFe] and [NiFe] hydrogenases (Fig. 3D). [FeFe]-hydrogenases were predominantly classified as type A3, while groups 2a, 3b, and 3d of [NiFe]-hydrogenases showed higher abundance (9.07–40.82%). [NiFe] group 3b was primarily affiliated with *Planctomycetota* (with a putative WL carbon fixation pathway) and *Pseudomonadota*. In contrast, [NiFe] group 3d was predominantly associated with *Bacteroidota* which contains a putative roTCA pathway (Figs. 2B, S1, and Table S8). This suggests the co-occurrence of specific hydrogenase types with distinct carbon fixation pathways may reflect a linkage between hydrogen metabolism and autotrophic energy acquisition and that possibly the stratified distribution of hydrogenases aligns with the vertical separation of redox conditions.

### Metagenomics-based energy contribution of electron donors

The contribution of potential electron donors varied across different depths, with energy likely being primarily provided by H_2_, NH_4_^+^, and S₂O₃²^−^ (Fig. 3). The contribution of H_2_ was likely highest at a depth of 749–752 cmbsf (~ 82.3%, Fig. 3F), coinciding with a higher abundance of putative [NiFe] hydrogenase group 2a and [NiFe] hydrogenase group 3b genes (Fig. 3D). This suggests that hydrogen-based energy metabolism may be preferentially utilized under deep, strongly reducing sediment conditions with limited electron acceptors. Genes encoding putative [NiFe] 3b and 3d hydrogenases were also more abundant at other depths (108–652 cmbsf) (Fig. 3D), and coincided with putative H_2_ donors and carbon fixation MAGs (Figs. 3F, S2), suggesting a potential link between hydrogen utilization and carbon fixation across multiple sediment layers. *Aerophobales* and *Bacteroidales* MAGs, which harbor putative WL and roTCA pathways, may acquire H₂-derived intermediate metabolites potentially produced by *Planctomycetota* (Fig. 3G), suggesting possible cross-feeding interactions based on hydrogen transfer among these lineages.

Metagenomic data suggest that NH_4_⁺ may represent a larger potential energy source at 15–24 cmbsf (29.32–35.28%) (Figs. 3F, S2). Only *Nitrososphaerales* MAGs containing genes encoding putative enzymes in the 3HP/4HB pathway and metabolism of NH₄⁺ (Fig. 2B, Table S2) showed higher abundance at the same depth (Fig. 2A). This depth-specific co-occurrence may reflect niche specialization under relatively oxic conditions. Oxidative metabolism of NH₄⁺ by *Nitrososphaeria* is supported by gene abundance, pathway distribution, and metabolite linkage patterns in this layer (Fig. 3G).

The contribution of energy from S₂O₃²^−^ oxidation may be significantly higher at 10–31 and 529–542 cmbsf, accounting for 48.4–60.7% of total energy (Fig. 3F), as estimated on the basis of reaction Gibbs free energy and predicted metabolic potential [46]. Based on MAG analysis, *Burkholderiales* are likely the core sulfur oxidative group in the deep sediment, producing and donating S²^−^ metabolites to *Dehalococcoidia* and *Planctomycetota* for carbon fixation. Such sulfur-mediated metabolic exchange may facilitate carbon fixation under stratified redox conditions. *Pseudomonadota*, which contains putative roTCA pathway, are likely the key species involved in sulfur oxidation (Fig. 2B, Table S2). Community structure analysis indicated that these key functional microbial groups showed significant abundance in the depths with high S₂O₃²⁻ concentrations (Fig. 2A, Table S2), consistent with potential coupling between sulfur oxidation and autotrophic carbon fixation.

### Correlation between physicochemical factors and functional genes

To elucidate the response mechanisms of microbial functional groups to habitat heterogeneity in the deep sediment, the covariation of functional genes involved in carbon fixation and carbon, nitrogen, sulfur metabolism across environmental factors, was analyzed (Fig. 3B and C). Oxidation-associated (*hao*, *nxrAB*) and reduction-associated (*narGHI*, *norBC*) genes showed a distinct vertical stratification in abundance distribution, indicating systematic variation in redox-sensitive metabolic potentials along sediment depth gradient. Within the nitrogen cycle (Fig. 3B), the oxidation-associated gene *hao* showed a negative correlation with NH₄^+^ (*r* = -0.58, *p*_FDR_ = 0.007; Table S11), which may reflect a link between nitrogen redox transformation and mineral-associated geochemical conditions. In contrast, the putative denitrification-related genes *nxrAB* and *narGHI* showed significant positive correlations with SiO₃²⁻ (both *r*s > 0.43, *p*_FDR_s < 0.05; Table S11), reflecting a possible link between nitrogen redox transformation and mineral-associated geochemical conditions. Notably, the putative hydrogenase gene *hya* and carbon monoxide oxidation gene *cooSF* showed significant positive correlations with NH₄⁺ (both *r*s > 0.51, *p*_FDR_s < 0.02; Table S11), indicating that hydrogen and carbon monoxide oxidation may co-occur with ammonium-rich conditions and potentially contribute to energy acquisition in these sediment layers. Regarding potential carbon fixation pathways (Fig. 3C), WL pathway genes (*acsA*, *acsB*, *CooS*) showed significant positive correlations with NH₄⁺ (all *r*s > 0.55, *p*_FDR_s < 0.02; Table S12), suggesting an association between reduced nitrogen availability and the prevalence of anaerobic carbon fixation pathways adapted to reduced conditions. Conversely, the *rbcS* gene of the CBB pathway showed a significant positive correlation with SiO₃²⁻ (*r* = 0.65, *p*_FDR_ = 0.002; Table S12), implying that CBB-based carbon fixation may be favored under distinct geochemical regimes associated with mineral inputs. After controlling for sampling depth, the validation results showed that all above correlations remained significant (Fig. S11).

### Traits of microbial carbohydrate-active enzymes in the deep sediment

**Fig. 4:**
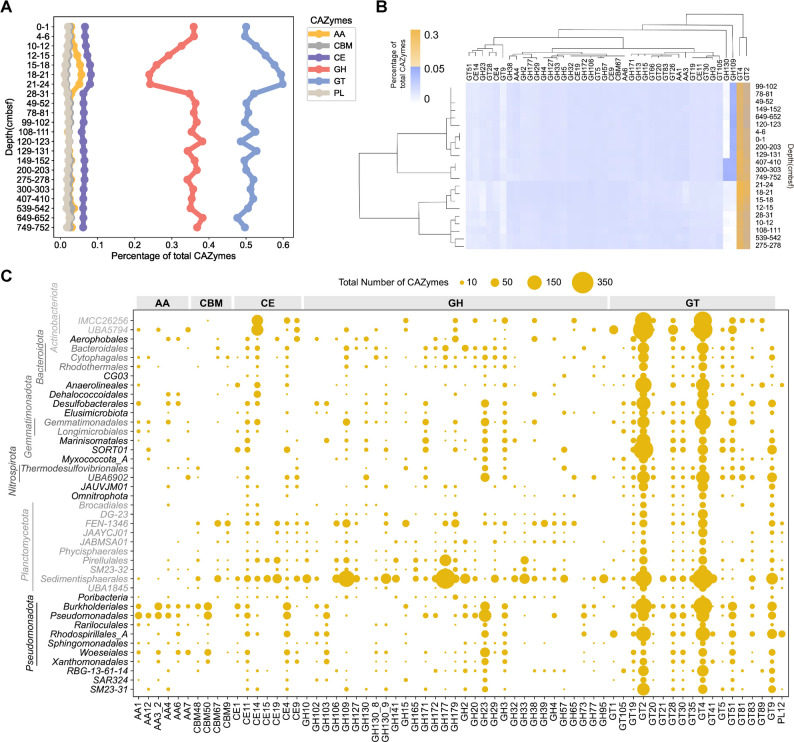
The utilization of carbohydrate-active enzymes (CAZymes) for microorganisms in the Mariana Trench deep sediment core. **A** Vertical distribution of putative CAZymes in the deep sediment. AA, auxiliary activity; CBM, carbohydrate-binding module; CE, carbohydrate esterase; GH, glycoside hydrolase; GT, glycosyltransferase; PL, polysaccharide lyase. **B** Vertical distribution of the CAZyme families within different depths of sediment. **C** Total count of CAZyme genes identified in carbon-sequestration MAGs across species

Putative carbohydrate-active enzymes (CAZymes) were identified from the metagenome and 1766 MAGs of the deep sediment using the dbCAN web server (Fig. 4A). Among the identified CAZy domains, predicted proteins including glycoside hydrolases (GH), glycosyltransferases (GT), and carbohydrate esterases (CE), were most abundant in the sediment. Conversely, the abundance of auxiliary enzymes (AA), carbohydrate binding modules (CBMs) and polysaccharide lyases (PL) was lower than 3.2% (Fig. 4A). The composition of CAZymes likely reflected the heterogeneity in carbohydrate utilization by microorganisms with different life histories across various depths (Fig. S2). Notably, CAZymes were more abundant and exhibited the highest diversity at 99–102 cmbsf, suggesting that this sediment horizon may provide relatively favorable conditions for carbohydrate turnover or accumulation of diverse organic substrates. Most of the MAGs (64%) harbored GT domains, which were distributed widely in the deep sediment, while GH domains were found in 104 MAGs (23.6%). CE and AA domains represented 6.6% and 3.2% of the CAZy domain content respectively, while CBM and PL together represented 4.3% of the domain content (Fig. 4B). Furthermore, the *Sedimentisphaerales* MAG was found to harbor the highest count of potential CAZy genes, which may indicate an enhanced capacity for utilizing diverse organic carbon sources which support metabolic flexibility, including their association with carbon fixation pathways described above (Fig. 4C).

## Discussion

### Spatial distribution of carbon fixation in the deep sediment

In this study, we uncovered a clear vertical stratification of microbial carbon fixation pathways across redox gradients in a Challenger Deep sediment core. Similar stratified patterns of microbial communities and metabolic potentials have been reported in other hadal trench sediments, suggesting that vertical redox-driven niche differentiation is a common feature of deep-sea trench ecosystems. Metagenomic and amplicon-based surveys of sediments from the Mariana, Japan, and Kuril-Kamchatka trenches [54], consistently revealed the enrichment of anaerobe-associated lineages such as *Planctomycetota* and *Chloroflexota* in deeper sediment layers. These taxa are frequently linked to anaerobic metabolism adapted to reduced sediment conditions and anaerobic carbon fixation strategies. Comparable vertical stratification has also been observed in non-hadal deep-sea sediments, such as in continental-slope and abyssal settings, where it largely reflects progressive organic-matter degradation and burial along diffusion-controlled geochemical gradients (e.g., increasing DIC and NH₄⁺ with depth) [55]. However, hadal trench environments differ from these systems in their geomorphology and sedimentary dynamics and therefore, have strongly reducing conditions relative to typical abyssal sediments [56, 57]. Consequently, while redox-driven stratification represents a general feature of marine sediments, the intensity of geochemical gradients and ecological filtering in hadal systems may be amplified by trench-specific deposition rather than depth alone. These observations are broadly consistent with the dominance of WL- and roTCA-associated taxa identified in the Challenger Deep sediment in this study, indicating that energetic constraints and redox conditions, rather than taxonomic identity alone, play a key role in shaping carbon fixation strategies in deep hadal sediments.

Rather than indicating carbon scarcity, the predominance of the WL pathway may reflect adaptation to redox-stratified subsurface conditions and efficient use of available electron donors. Measured TOC values remain relatively stable throughout the sediment column (0.32–0.49%; Table S5), implying a persistent supply of organic carbon even at depth. Such TOC levels are comparable to those reported for many hadal sediments and do not suggest strong carbon depletion with burial. Hadal trench sediments are increasingly recognized as depositional centers of organic matter with elevated microbial activity and shallow oxygen penetration depths, indicating active mineralization rather than simple energy limitation [58]. Overall, the WL, rGly, and roTCA pathways displayed significantly higher abundance compared to other pathways. This pattern may reflect their metabolic compatibility with steep redox gradients and the progressive depletion of electron acceptors that typify subsurface trench sediments, rather than just their lower ATP requirements. Such redox-driven structuring of microbial metabolism is consistent with observations that hadal sediments often exhibit high organic carbon inventories but rapid oxygen consumption and intense anaerobic processing, resulting in strong vertical redox zonation that shapes microbial functional strategies [58].

The WL, roTCA and rGly pathways were the dominant predicted carbon fixation pathways in the deep sediment, exhibiting a higher abundance across the depth of sediment. They have been shown to be the key CO₂ fixation pathways in anaerobic or microaerophilic microorganisms [12, 14], requiring substantially lower energy input compared to aerobic counterparts like the CBB cycle, the 3HP cycle, and the 3HP/4HB cycle [11]. In the South China Sea slope sediments, the correlation between DIC/NH₄⁺ concentrations and the vertical distribution of carbon fixation pathways was strong, explaining 66.7–100% of the variation in key gene families for WL, rTCA, rGly, and roTCA pathways [55]. In contrast, previous studies [59] focusing on hadal seawater or surface sediments have more frequently reported the dominance of rTCA- or CBB-based carbon fixation, particularly among nitrifying and sulfur-oxidizing microorganisms. This apparent discrepancy is likely attributable to higher oxygen availability and ammonium fluxes in seawater or shallow sediment layers, which relax redox constraints, increase availability of high-potential electron acceptors and favor aerobic or microaerophilic autotrophic pathways. The predominance of WL and rGly pathways observed here therefore likely reflects the more strongly reducing conditions and limited availability of high-potential electron acceptors in deeper sediment horizons of the Challenger Deep. Accordingly, the WL and roTCA pathways were mainly distributed in hypoxic sediment of the Challenger Deep, where energy conservation and redox constraints strongly governed microbial survival strategies, as their key enzymes were highly sensitive to oxygen [60].

*Planctomycetota* MAGs reliant on WL pathways were predominant in the hadal deep sediment [33], in contrast to marine sediments from other regions, where *Pseudomonadota* dominated carbon fixation in Bohai Sea sediments and cold seep ecosystems [24, 61]. In particular, *Sedimentisphaerales* and *Aerophobota* MAGs were significantly enriched in the anaerobic layers (100–752 cmbsf), which were characterized as NH₄⁺-rich [33]. This indicated a strong correlation between WL pathway genes and NH₄⁺, which can be mechanistically explained by redox-driven environmental selection favoring WL-based anaerobic carbon fixation in deep sediment. Interestingly, *Aerophobota* were also detected at notable abundance in the uppermost sediment layers, which is unexpected for typical deep-sediment anaerobes. This vertical distribution likely reflects episodic sedimentation events where ancient anaerobic communities, including *Aerophobota*, were transported from slope regions to both surface and deep sediments, allowing them to persist even in layers where oxygen conditions would normally limit their growth [33]. Overall, these observations suggest that the vertical distribution of *Aerophobota* in the Challenger Deep sediment results from the combined effects of episodic sedimentation delivering external anaerobic populations and redox-driven environmental selection favoring WL pathway-mediated carbon fixation. Although *Dehalococcoidia* MAGs have been annotated as lacking a defined electron sink, they may derive energy from organohalide compounds via the process of dehalogenation [62]. In addition, previous studies have shown that *Gemmatimonadota* play a pivotal role in sulfur, nitrogen, and oxygen metabolism within coastal sediments [63–65]. In this study, *Gemmatimonadota* MAGs in the oxic layer (15–27 cmbsf) were apparently able to drive carbon fixation through coupling with sulfate (*sat*) and nitrate (*nrfAH*) reduction processes.

The metabolic potential inferred for the *Pirellulales* MAGs is consistent with emerging genomic insights into this lineage. Previous studies indicate that members of *Pirellulales* mainly encode the assimilatory sulphate reduction (ASR) pathway, which supports sulfur incorporation into biomass rather than dissimilatory sulphate reduction linked to energy conservation [66]. Accordingly, sulfur metabolism genes detected in our MAGs likely reflect biosynthetic sulfur assimilation. In addition, *Planctomycetota* are widely predicted to generate energy via glycolysis and to possess the capacity for carbohydrate fermentation [66], suggesting that the fermentative traits inferred here are compatible with known metabolic features of this phylum. Together, these observations indicate that *Pirellulales* in our sample may contribute to organic matter turnover and elemental cycling while participating in carbon fixation, pointing to a potentially broader ecological role for this group in anaerobic environments.

The roTCA cycle in deep sediment was dominated by obligate anaerobes *Marinisomatota* [67] and *Actinomycetota*. *Marinisomatota* likely primarily used hydroxylamine as an electron donor and sulfate as an electron acceptor, whereas *Actinomycetota* likely primarily oxidize sulfide and nitrite as electron donors for energy production. *Marinisomatota* facilitate microbial interactions via sulfur reduction, nitrogen fixation, denitrification, and mixed-acid fermentation [67, 68], while *Actinomycetota* rely on sulfide oxidation for energy metabolism [69]. Consistent with the preferential utilization of the energetically frugal roTCA cycle for carbon fixation by *Pseudomonadota* under hypoxic conditions [14], *Burkholderiales* potentially supported roTCA carbon fixation through sulfur oxidation (*fccAB*, *sox*) in deep sediment [70, 71]. *Bacteroidia* MAGs relied on the roTCA pathway, potentially utilizing H_2_ as an electron donor (Fig. 3E).

**Fig. 5:**
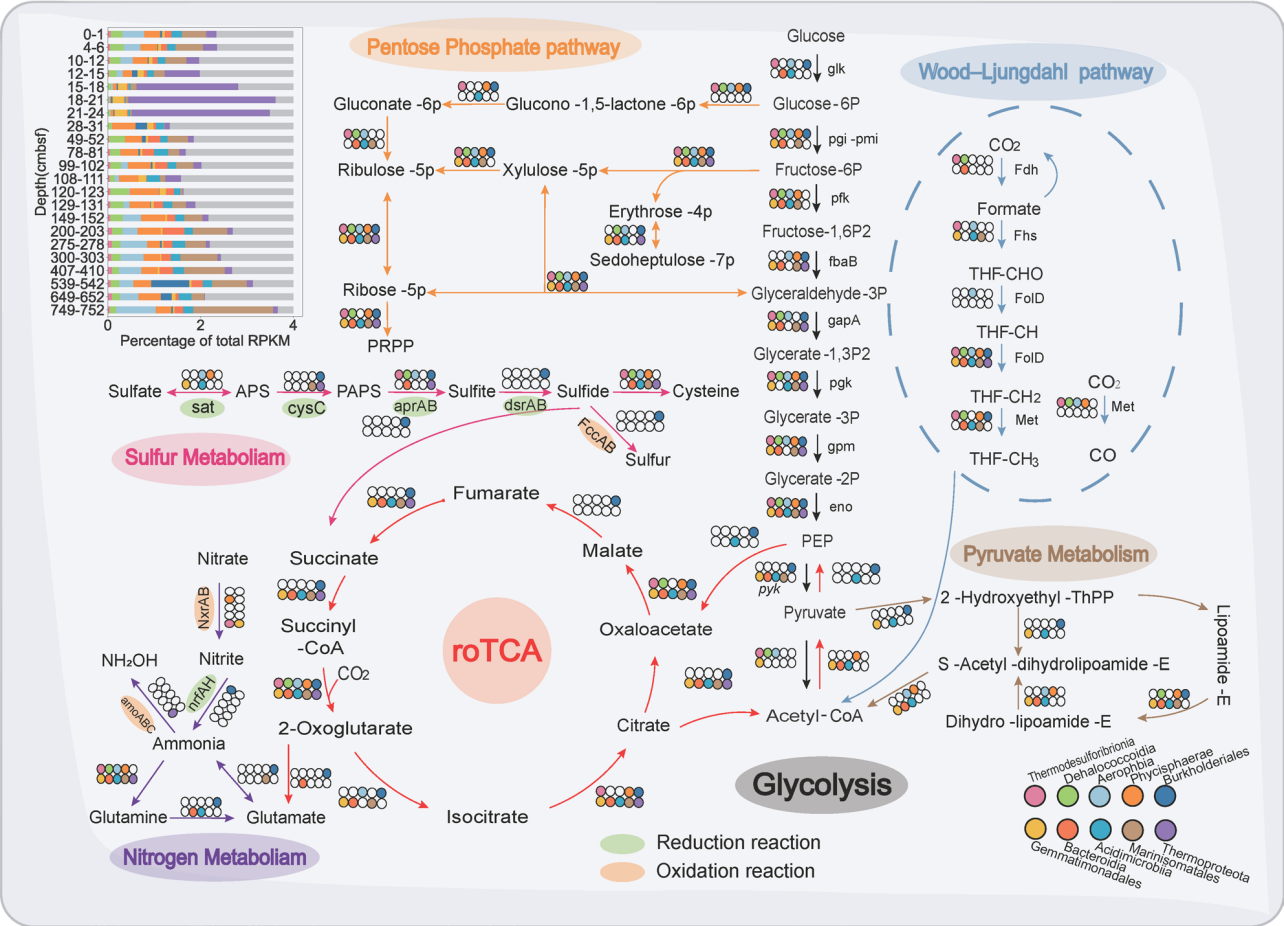
The capabilities of inferred metabolism in ten representative autotrophic MAGs. The existence of predicted metabolic pathways and associated genes potentially driving carbon fixation identified in these MAGs was visually denoted with colored dots, whereas white dots indicated the absence of pathway or gene. Targeted pathways encompassed carbon fixation, sulfur metabolism, nitrogen metabolism, and carbohydrate degradation. The ellipse colors corresponded to the reaction types: oxidation (orange) and reduction (green)

Four archaeal MAGs affiliated with ammonia-oxidizing archaea *Nitrososphaeria* harbored the complete 3HP/4HB pathway and were the major group mediating this pathway, mainly found at 15–27 cmbsf (Fig. 5). Ammonia-oxidizing archaea have been widely recognized as major contributors to autotrophic carbon fixation in hadal seawater and surface sediments, where ammonium oxidation provides a stable energy source under oxic or suboxic conditions [59]. The restriction of 3HP/4HB-harboring *Nitrososphaeria* to relatively shallow sediment layers therefore likely reflect depth-dependent niche partitioning driven by oxygen availability. With increasing sediment depth and decreasing oxygen availability, archaeal nitrifiers are gradually replaced by bacterial lineages utilizing anaerobic carbon fixation pathways. In addition to ammonia oxidation and carbon fixation, these MAGs contained genes related to sulfide oxidation, consistent with recent genomic studies suggesting that some *Nitrososphaeria* populations may possess broader metabolic versatility and participate in sulfur transformations in marine sediments [72]. Such metabolic flexibility may enhance their ecological competitiveness in shallow sediment horizons where oxygen gradients intersect with reduced sulfur compounds.

Studies from the Mariana Trench have demonstrated that nitric oxide reduction and related denitrification processes occur in surface sediment and seawater, although these processes are primarily attributed to *Pseudomonadota* [59]. The detection of *norBC* genes in our *Nitrososphaeria* MAGs therefore raises the possibility that this archaeal lineage may also contribute to local NO turnover, albeit likely at lower abundance than bacterial denitrifiers. Alternatively, these genes may reflect horizontal gene transfer or binning uncertainty, and their physiological relevance remains to be experimentally validated. If functional, this metabolic combination would suggest that *Nitrososphaeria* contribute not only to ammonia-driven carbon fixation but also to coupled nitrogen-sulfur cycling in hadal surface sediments.

In addition, based on our results, many of the carbon-fixing microorganisms across diverse phyla could potentially be mixotrophic, since they possessed genes enabling utilization of a variety of organic carbon compounds, primarily polymeric substrates, with GH and GT playing key roles [73]. Previous geochemical and microbiological investigations have shown that hadal trenches act as effective depocenters for organic matter [39], resulting in the accumulation of labile, nitrogen-rich organic matter that may support microbial communities with potential for mixotrophy in trench sediments. Consistent with this framework, the widespread detection of CAZy genes among autotrophic MAGs in this study supports the view that mixotrophy may represent an ecologically advantageous strategy in deep hadal sediments. Mixotrophy allows organisms to combine autotrophic and heterotrophic pathways to exploit both inorganic and organic resources, providing metabolic flexibility under resource‑limited or fluctuating conditions [74]. Recent metagenomic studies also indicate that mixotrophic capacities may be widespread in deep, aphotic marine environments, suggesting a similar ecological relevance in hadal sediments [75]. Furthermore, members of the *Sedimentisphaerales* contained the largest fraction of CAZy genes in the deep sediment [76]. Notably, the autotrophs *Xanthomonas* and IMCC26256 predominantly obtained energy via fermentation, which presumably provides the energy necessary to drive carbon fixation via the CBB cycle and roTCA pathway, respectively.

### Distinct energy donation to carbon fixation in the deep sediment

Consistent with observations in the South China Sea slope sediments [55], energy sources such as H₂ and NH₄⁺ can strongly influence the distribution of anaerobic carbon fixation pathways, as shown by the preferential enrichment of *Planctomycetota*, *Aerophobales* and *Bacteroidales* in hypoxic and ammonium-rich layers in our dataset. H_2_ was hypothesized as the main energy source in the deep sediment, contributing to carbon fixation in *Planctomycetota*, *Aerophobales* and *Bacteroidales* (Fig. 3E). *Planctomycetota* has been shown to utilize hydrogen as an energy source [25] while *Actinomycetes* [77] and *Desulfobacteraceae* [78] can utilize NiFe and FeFe hydrogenases to mediate the WL pathway in hot spring sediments. *Bacteroidales* have been shown to utilize the roTCA pathway driven by [NiFe] 3d hydrogenases [25, 79]. Moreover, *Aliihoeflea* could potentially mediate the CBB pathway through NiFe hydrogenases in the sediment. Anaerobic CO oxidation may also be an alternative energy source [80].

NH₄⁺, another key energy donor, accounted for 1–35% of the energy contribution, supporting alternative chemolithoautotrophic groups across depths of the sediment. Shi et al. [55] also reported strong correlations between NH₄⁺ concentrations and the abundance of WL- and rGly-associated genes, suggesting that ammonium availability may modulate carbon fixation strategies in deep sediments. Under the metabolic theory framework, ammonium functions not merely as a nutrient, but as a redox-active energy source that supports chemolithoautotrophic carbon fixation when organic substrates are scarce. The key WL pathway group, *Sedimentisphaerales*, could utilize NH_2_OH producing NO₂⁻ to support their autotrophic processes, with NH₂OH derived from ammonium oxidation (Fig. 5) by other microorganisms, suggesting that the WL pathway was highly associated with the concentration of NH₄⁺ (Fig. 3C). In contrast, *Nitrososphaerales*, which contained most 3HP/4HB pathways, replaced *Sedimentisphaerales* at 12–15 cmbsf, and was also likely capable of retaining electrons from low levels of NH_4_^+^ to support their autotrophic processes [33, 81]. This taxonomic replacement likely reflects differences in energetic efficiency and substrate affinity, with distinct autotrophic pathways being favored under varying ammonium concentrations and redox conditions. *Burkholderiales* were inferred to mediate the roTCA pathway coupled with sulfur oxidation [82–84], using sulfide as the electron donor and transferring electrons to available respiratory electron acceptors (Fig. 5), indicating that sulfur-oxidizing bacteria played a vital role in carbon fixation in the sediment.

### Vertical distribution and plasticity of microbial carbon fixation in the sediment driven by redox condition and matter availability

**Fig. 6:**
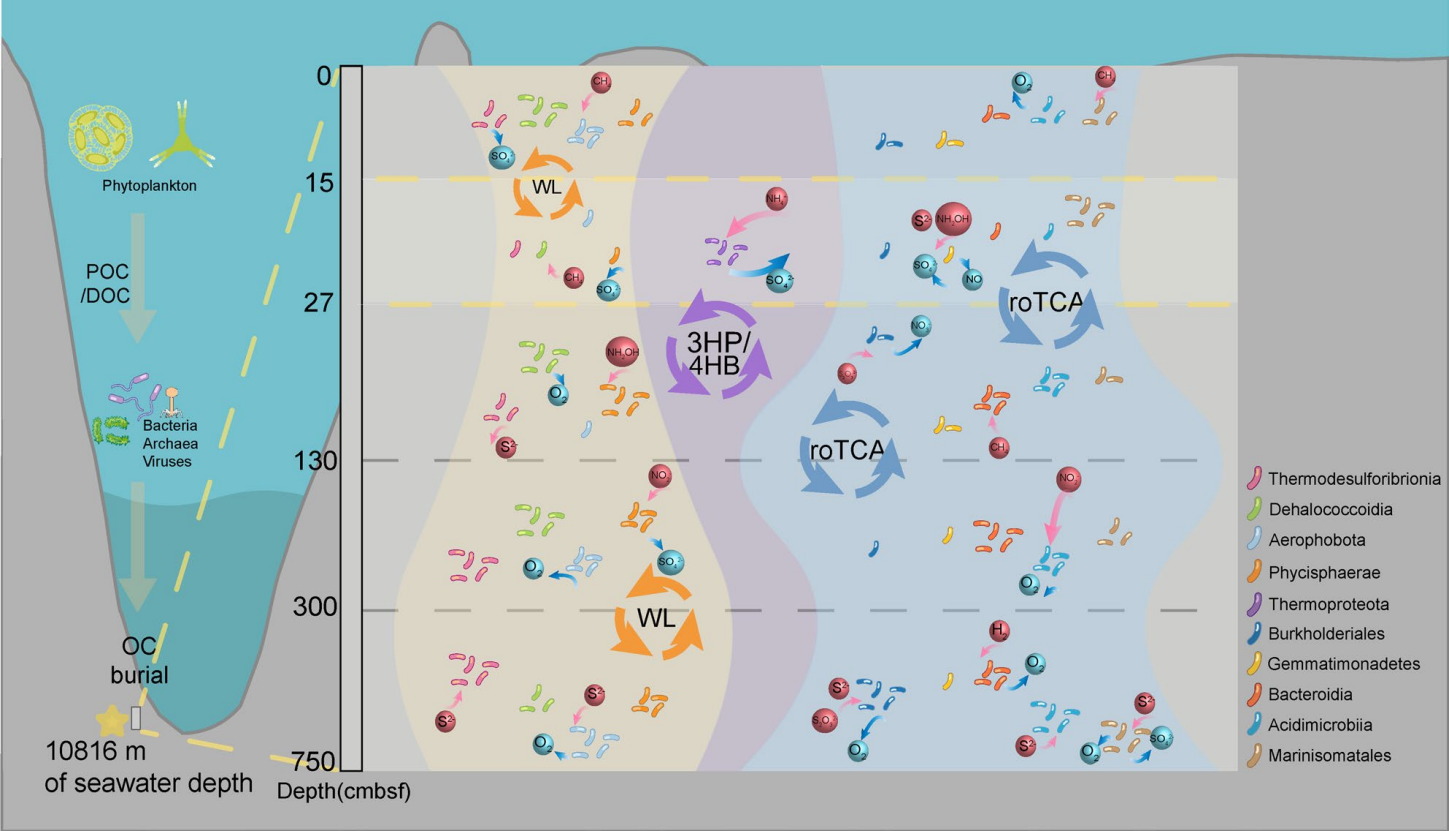
Traits of autotroph-associated electron donors and acceptors, potential metabolite transfers in deep sediment. The beige, light purple, and light blue backgrounds represent the WL, 3HP/4HB, and roTCA carbon fixation pathways, respectively. Background width corresponds to the average abundance of carbon fixation pathway-specific marker genes at each sediment depth. The highlighted region (15–27 cmbsf) denotes the oxidative environment

According to redox tower and niche differentiation theories, microbial communities are expected to exhibit pronounced vertical stratification according to the strong vertical redox gradients [85]. This was the case in this study where carbon fixation potential shifted in response to changing redox conditions and matter availability (Fig. 6). From 0–12 cmbsf, carbon fixation potential decreased with increasing depths, due to efficient material exchange and the episodic deposition of older material from the slope [33]. Nitrifying *Nitrosopumilales* and sulfur-oxidizing bacteria were more abundant at 15–27 cmbsf, indicating that the oxidizing environment likely provided suitable conditions for their proliferation. As the oxygen content gradually decreased below 28 cmbsf, the environment transitioned into a microoxic state. This redox transition marks a critical ecological boundary where availability of high-potential electron acceptors declines and niche differentiation intensifies. In this region, microbial hydrogen-based autotrophs increasingly dominated the carbon fixation processes, and it is likely that the WL and roTCA pathways became increasingly coupled with nitrogen and sulfur metabolism. Such coupling reflects adaptive niche partitioning, whereby autotrophs exploit alternative electron donors and acceptors to sustain carbon fixation under increasing redox constraints and limited oxidant availability. In the microoxic region at 539–542 cmbsf, the abundance of sulfur-oxidizing bacteria sharply increased. The chemolithoautotrophic microbes related to sulfur oxidation metabolism (such as *Epsilonproteobacteria* and *Gammaproteobacteria*) primarily fix CO₂ through the rTCA or CBB cycles in anaerobic or microoxic environments [86]. The sulfur-oxidizing bacteria *Burkholderiales*, likely utilizing the energy-efficient roTCA pathway and relying on both hydrogen and reduced sulfur compounds as energy sources, reached peak abundance at 749–752 cmbsf. The dominance of sulfur-oxidizing autotrophs employing energy-efficient pathways at depth suggests strong thermodynamic filtering, where only metabolism capable of maximizing energy conservation remains competitive.

## Conclusions

Comparisons with previous hadal sediment studies suggest that, while the relative importance of specific carbon fixation pathways may vary among trench systems, the overarching ecological controls imposed by redox gradients and organic matter availability are consistent. The Challenger Deep therefore represents an extreme example of hadal environments, where intense redox stratification strongly influences the distribution of anaerobic and energetically efficient carbon fixation strategies. This structuring reflects adaptation to steep vertical redox gradients and progressive depletion of electron acceptors, consistent with high microbial activity and rapid oxygen consumption in hadal sediments. These patterns underscore the high adaptability and metabolic plasticity of deep sediment autotrophs, providing empirical support for ecological theories predicting niche differentiation and metabolic optimization along steep environmental gradients.

## Supplementary Information

Below is the link to the electronic supplementary material.

Supplementary Material 1

Supplementary Material 2

## Data Availability

The metagenome data and metagenome-assembled genomes data are from our recent work [36] and deposited in the NCBI database under the accession number PRJNA957232 and PRJNA957236. The 1766 MAGs were deposited in eLMSG (an eLibrary of Microbial Systematics and Genomics, https://www.biosino.org/elmsg/index) under accession numbers LMSG_G000011653.1-LMSG_G000013418.1. The 544 carbon fixation-related MAGs were deposited in NCBI database under the accession number PRJNA1446219 and PRJNA1446231.

## Abbreviations

MAGs: Metagenome-assembled genomes
WL: Wood-Ljungdahl
roTCA: Reverse oxidative TCA
3HP/4HB: 3-hydroxypropionate/4-hydroxybutyrate
CBB: Calvin-Benson-Bassham
rGly: Reductive glycine pathway
DC/4HB: Dicarboxylate/4-hydroxybutyrate cycle
rTCA: Reductive tricarboxylic acid cycle
3HP: 3-hydroxypropionate bicycle
TOC: Total organic carbon
TN: Total nitrogen
OC: Organic carbon
FDR: False discovery rate
CAZymes: Carbohydrate-active enzymes
GH: Glycoside hydrolases
GT: Glycosyltransferases
CE: Carbohydrate esterase
AA: Auxiliary enzymes
CBMs: Carbohydrate binding modules
PL: Polysaccharide lyases
