# Supplementary material for: Vertical distribution and metabolic diversity of autotrophic microbes in the deep sediment of the challenger deep
Source: Environ Microbiome. 2026 May 12;21:86. doi: 10.1186/s40793-026-00908-5 (PMC13335318; doi:10.1186/s40793-026-00908-5)
Supplement: Supplementary file 1 — Supplementary Material 1 [file 40793_2026_908_MOESM1_ESM.pdf]

Supplementary Material

Vertical distribution and metabolic diversity of autotrophic microbes in the deep sediment of the Challenger Deep

Jiahua Zhou[1,3†], Haojin Cheng[1,3†], Yulin Zhang[1,3], Tianhang Liu[4], Xing Chen[1,3], David J. Lea-Smith[5], Jonathan D. Todd[1,5,6], Jiwen Liu[1,2,3], Xinxin He[1,3], Ronghua Liu[1,3], Xiao-Hua Zhang[1,2,3*]

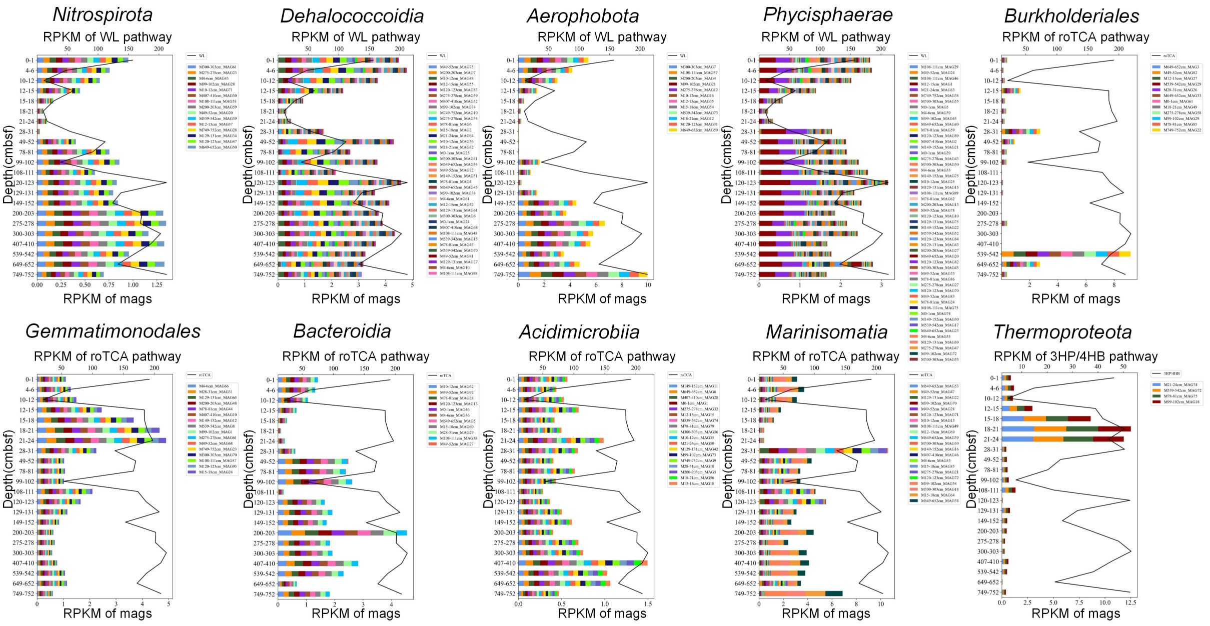

Fig. S1 Vertical distributional patterns of carbon fixation MAGs across dominant taxa.

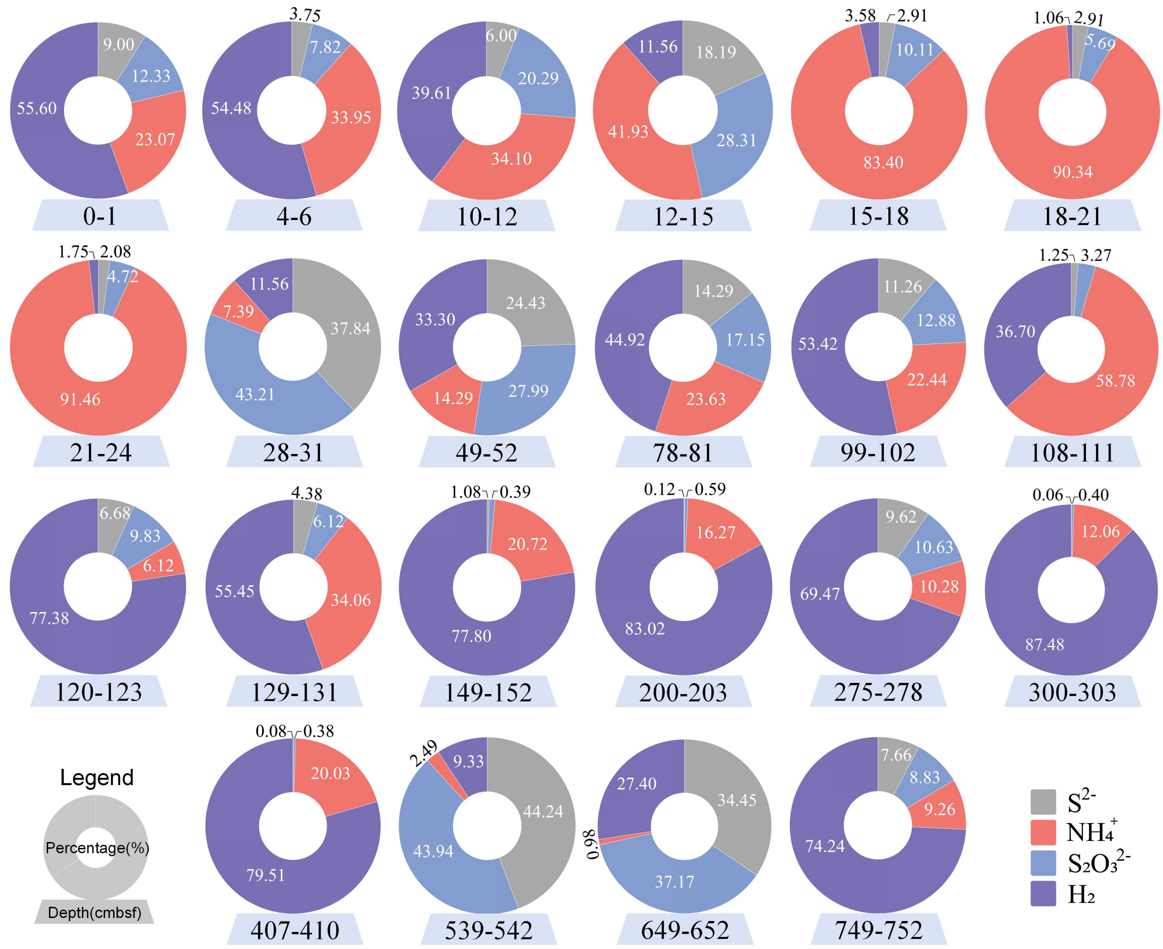

Fig. S2 Energy contribution estimation based on autotrophic MAGs. Energy contribution donated by varied electron donors was inferred at the metagenome-assembled genome (MAG) level, reflecting the genomic metabolic potential of individual autotrophic populations along sediment depth (cmbsf, centimeters below seafloor). The contribution ratio of electron donor species was calculated based on the presence and completeness of corresponding metabolic pathways in autotrophic MAGs and summarized for individual environments respectively. The resulting data are provided in Supplementary Table S7.

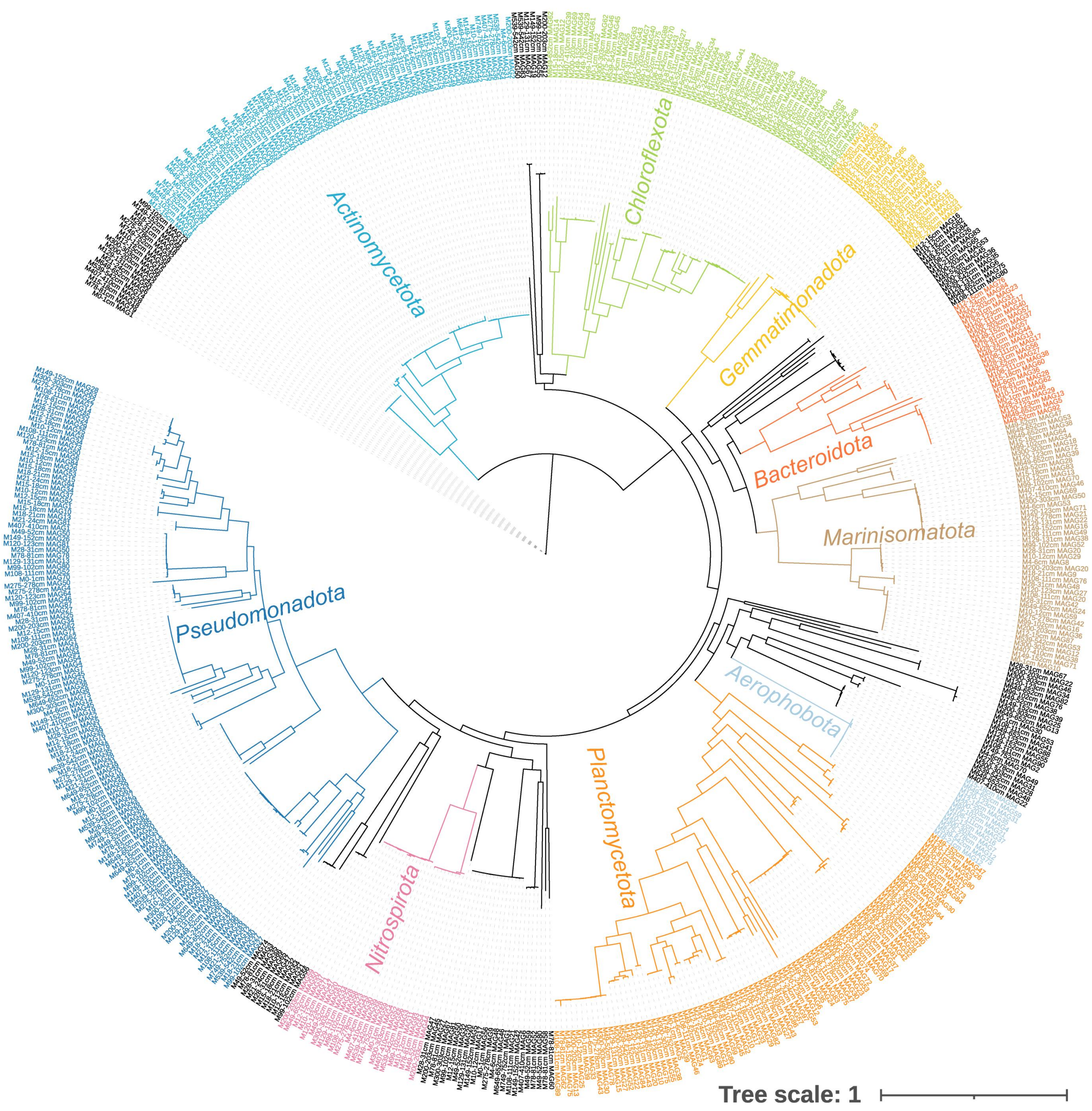

Fig. S3 Phylogenetic tree of 539 bacterial autotrophic MAGs based on 120 single-copy proteins. The phylum of MAGs was represented by colored branches.

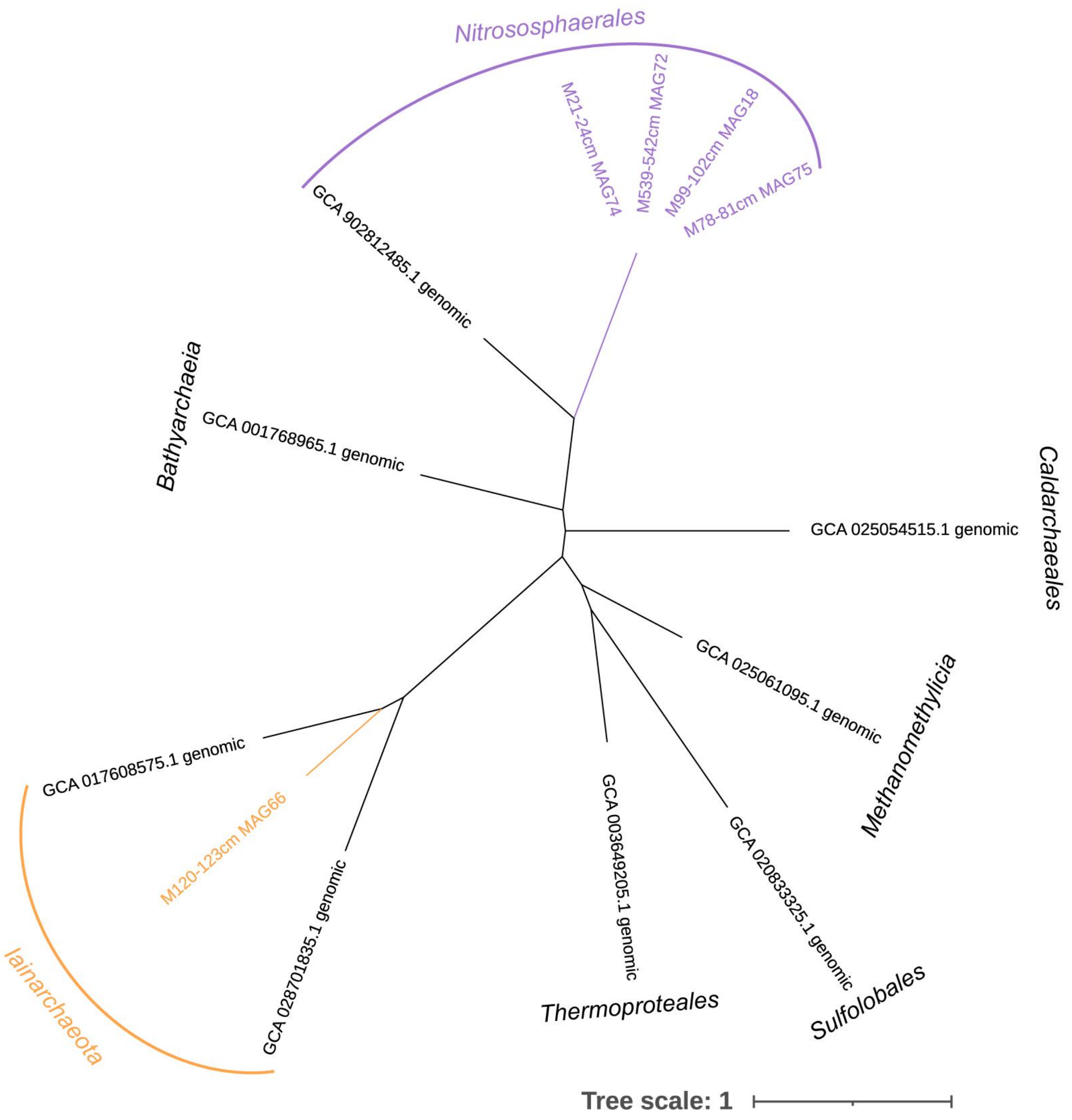

Fig. S4 Phylogenetic analysis of 5 archaeal MAGs based on 122 single-copy protein-coding marker genes.

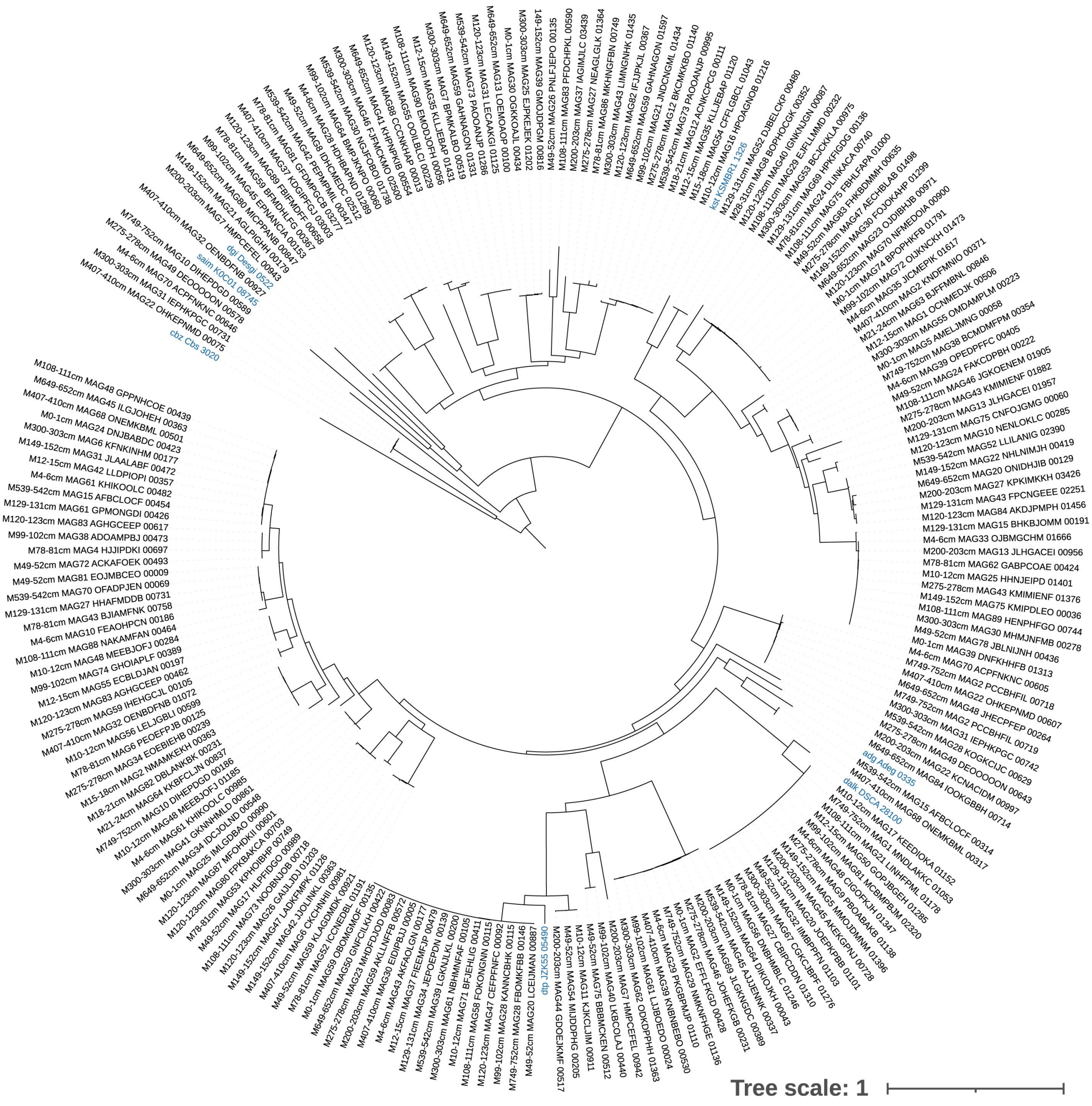

Fig. S5 Phylogenetic tree of the *acsA* (K00198) protein within MAGs. K00198 encodes the carbon monoxide dehydrogenase catalytic subunit, a signature enzyme of the Wood – Ljungdahl pathway. Standard proteins in the tree are labelled in blue based on KEGG annotation results (WP_012059212.1, WP_221169328.1, WP_006523451.1, WP_099324593.1, WP_015738374.1, WP_155316982.1, WP_207105979.1).

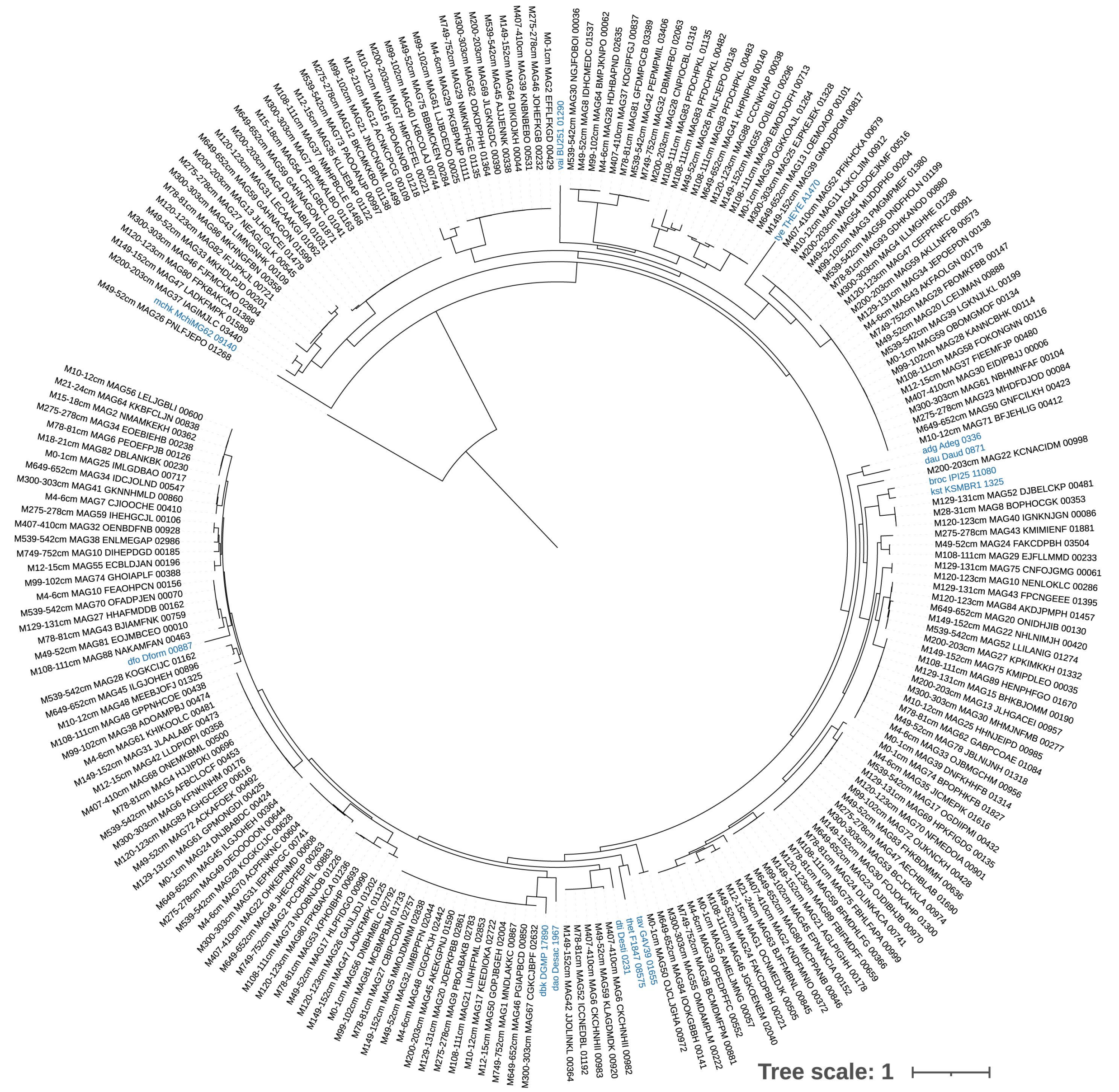

Fig. S6 Phylogenetic tree of the *acsB* (K14138) protein within MAGs. K14138 encodes the acetyl-CoA synthase, a signature enzyme of the Wood – Ljungdahl pathway. Standard proteins in the tree are labelled in blue based on KEGG annotation results (WP_221058095.1, WP_012546472.1, WP_015738375.1, WP_012301970.1, WP_007222621.1, WP_099324592.1, WP_013163059.1, WP_013163059.1, WP_014808136.1, WP_013706909.1, WP_003418359.1).

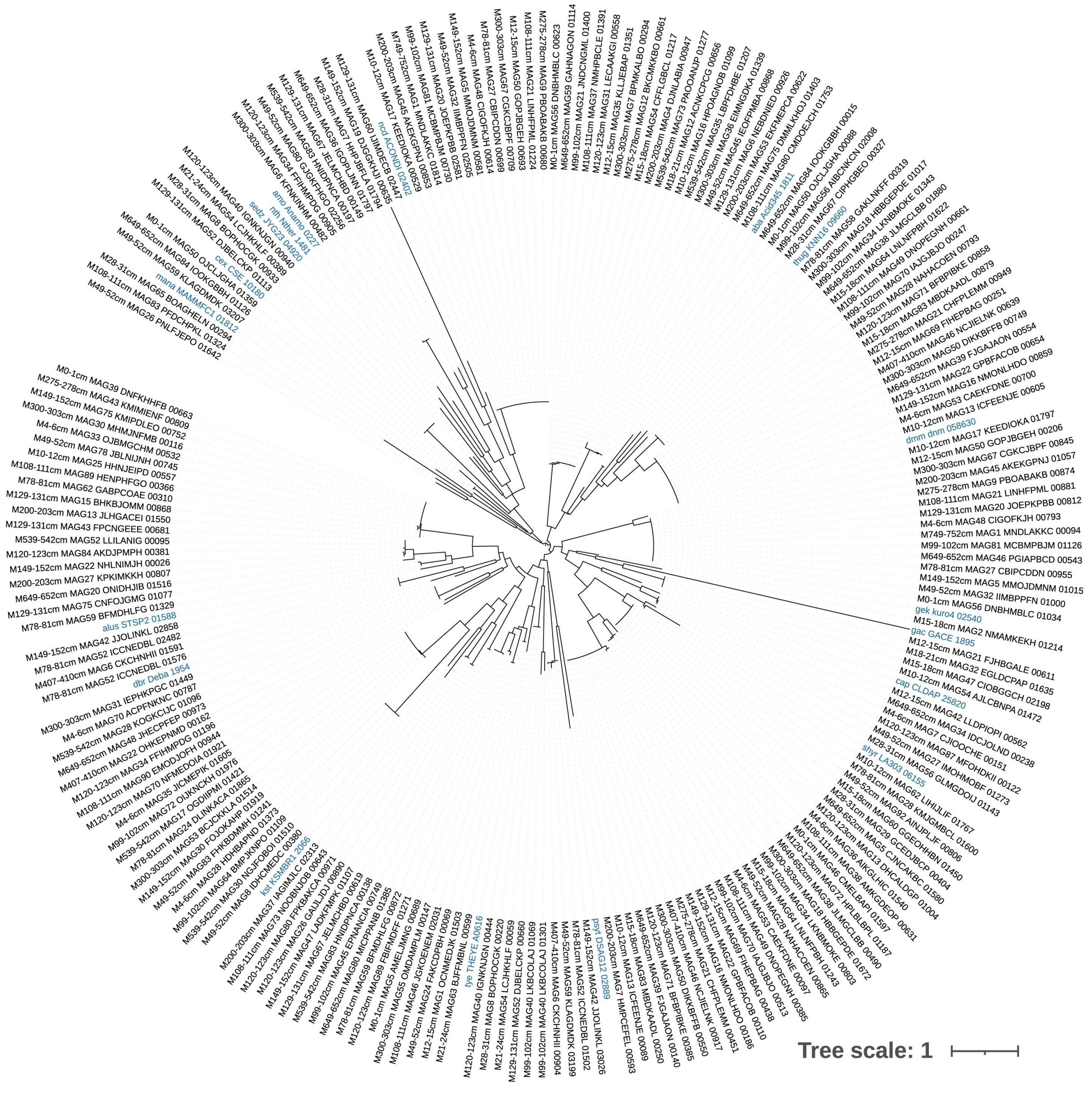

Fig. S7 Phylogenetic tree of the *korD* (K00176) protein within MAGs. K00176 encodes the 2-oxoglutarate ferredoxin oxidoreductase subunit delta, a signature enzyme of the reversed oxidative TCA cycle. Standard proteins in the tree are labelled in blue based on KEGG annotation results (WP_126308203.1, WP_014453546.1, WP_019227052.1, WP_012447936.1, WP_014806127.1, WP_241078795.1, WP_011522614.1, WP_006848322.1, WP_207678273.1, WP_003420706.1, WP_048092953.1, WP_014433851.1, WP_010259342.1, WP_147663976.1, WP_012545602.1, WP_099325264.1, WP_013258760.1, WP_146661423.1).

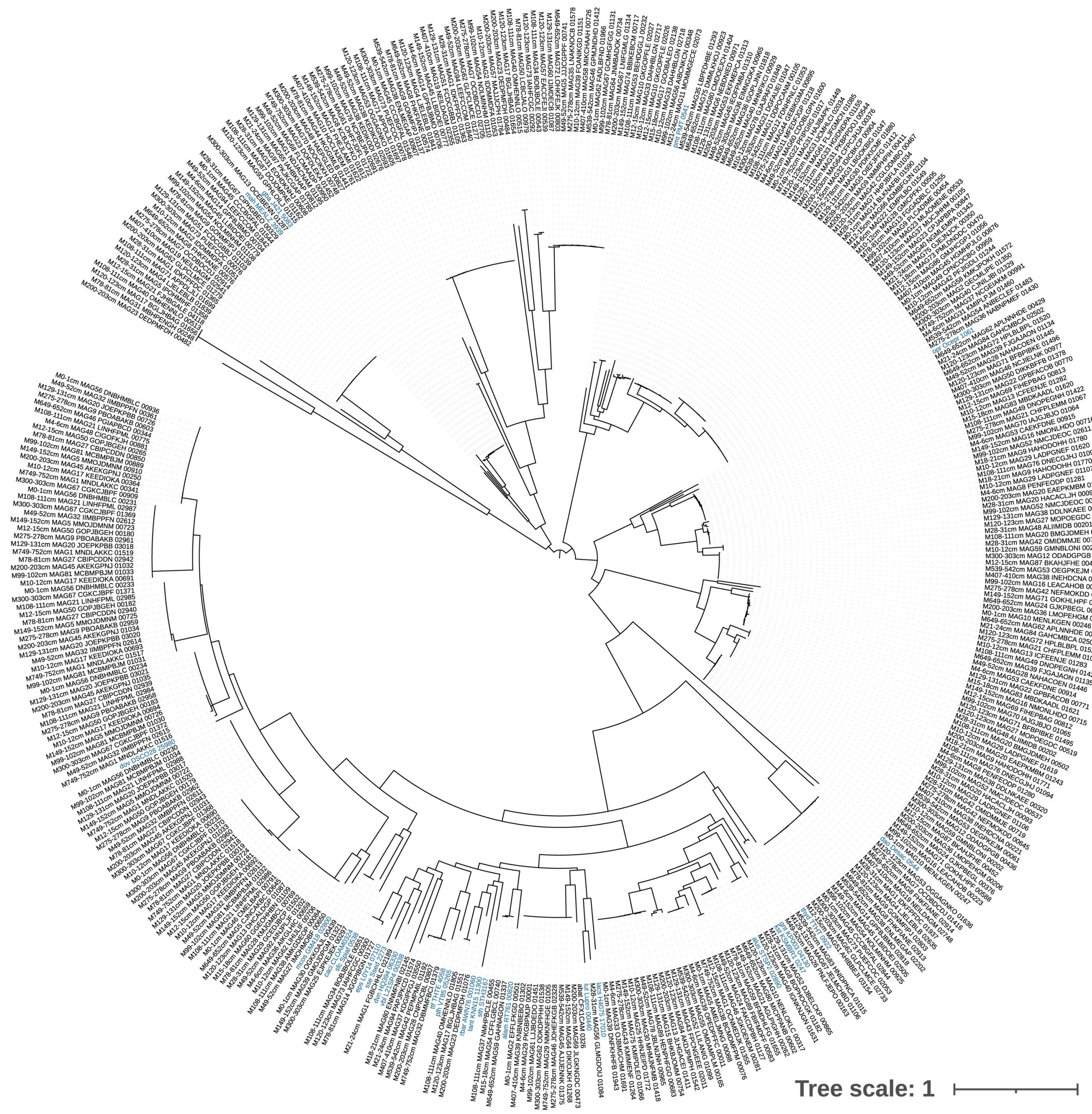

Fig. S8 Phylogenetic tree of the *por* (K03737) protein within MAGs. K03737 encodes the pyruvate - ferredoxin/flavodoxin oxidoreductase, a signature enzyme of the reversed oxidative TCA cycle. Standard proteins in the tree are labelled in blue based on KEGG annotation results (WP_025413949.1, WP_011129920.1, WP_013457688.1, WP_013705543.1, WP_201328828.1, WP_015721537.1, WP_099323687.1, WP_146663358.1, WP_016195742.1, WP_013306254.1, WP_218112809.1, WP_011197280.1, WP_015717204.1, WP_012122506.1, WP_068411584.1, WP_052882931.1, WP_013768846.1, WP_190262550.1, WP_012142617.1, WP_005964713.1, WP_014454638.1, WP_015424090.1, WP_016777118.1, WP_155322582.1).

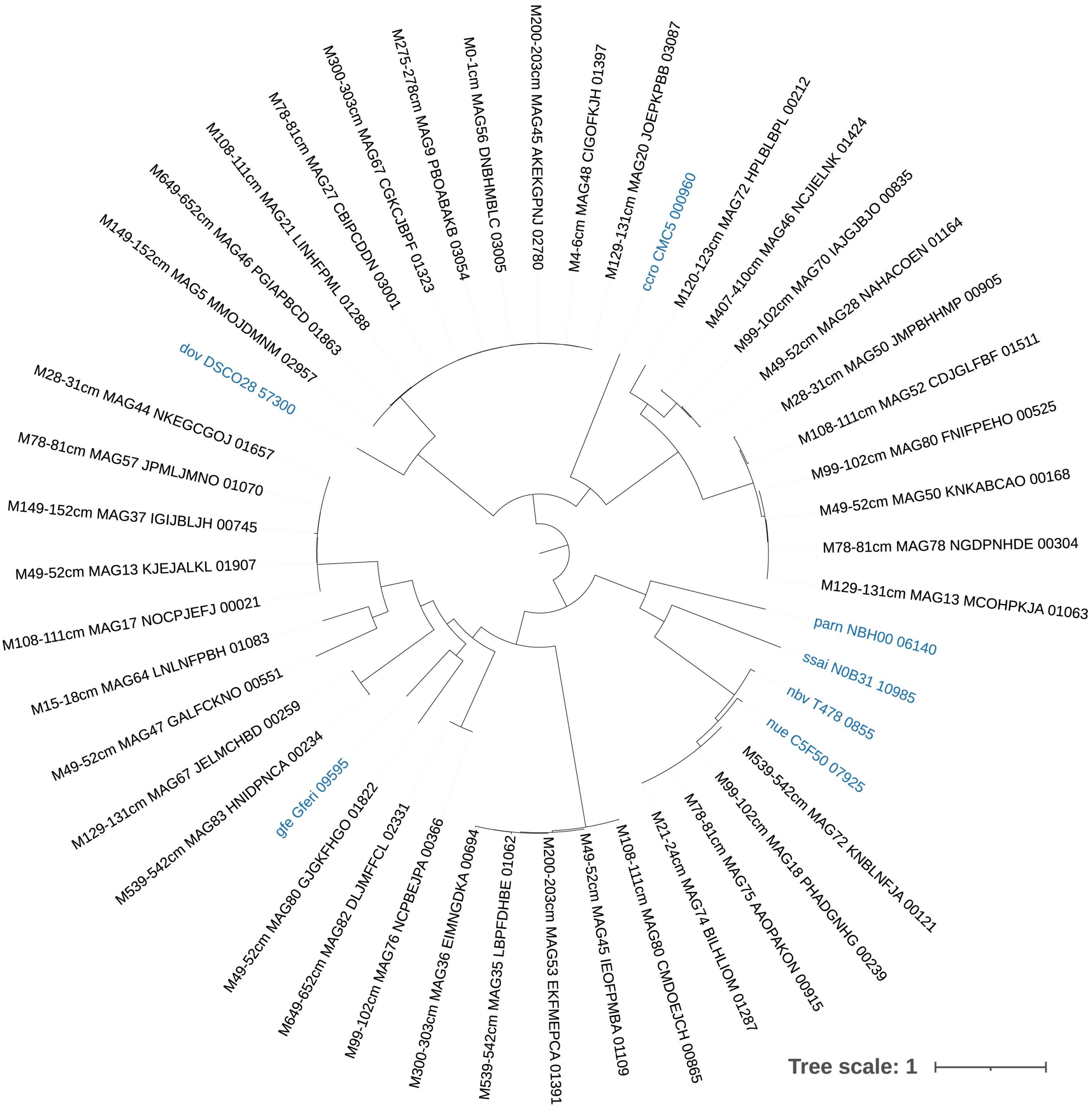

Fig. S9 Phylogenetic tree of the K15016 protein within MAGs. K15016 encodes the 3-hydroxyacyl-CoA dehydrogenase, a signature enzyme of the 3-hydroxypropionate/4-hydroxybutyrate cycle.. Standard proteins in the tree are labelled in blue based on KEGG annotation results (WP_010877531.1, WP_012829691.1, WP_254572470.1, WP_260643913.1, WP_048106914.1, WP_179370863.1, WP_069975890.1).

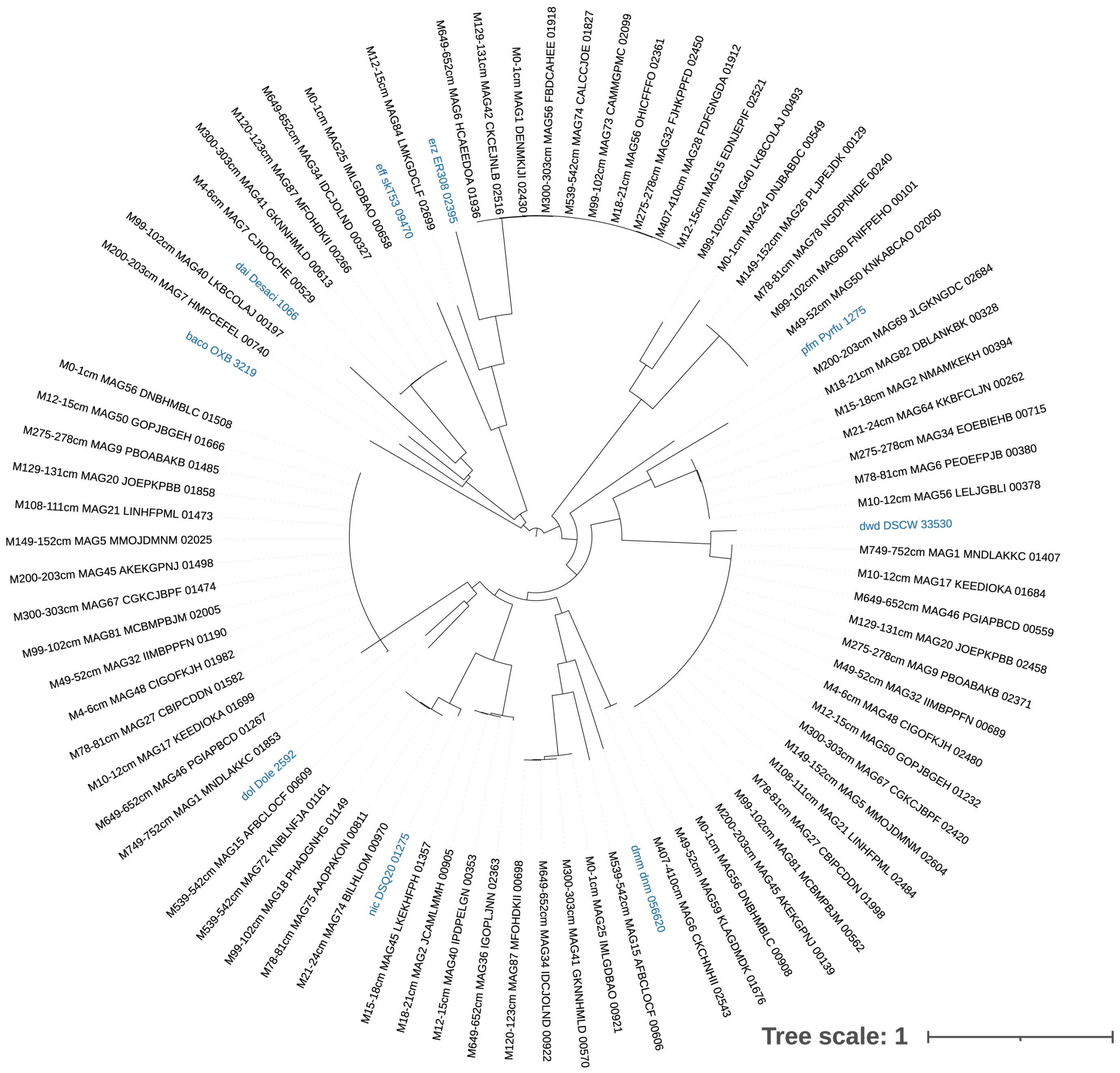

Fig. S10 Phylogenetic tree of the *abfD* (K14534) protein within MAGs. K14534 encodes the 4-hydroxybutyryl-CoA dehydratase, a signature enzyme of the 3-hydroxypropionate/4-hydroxybutyrate cycle. Standard proteins in the tree are labelled in blue based on KEGG annotation results (WP_052484114.1, WP_014826113.1, WP_052846143.1, WP_131153527.1, WP_014026811.1, WP_048064616.1, WP_207678151.1, WP_179365767.1, WP_012176007.1).

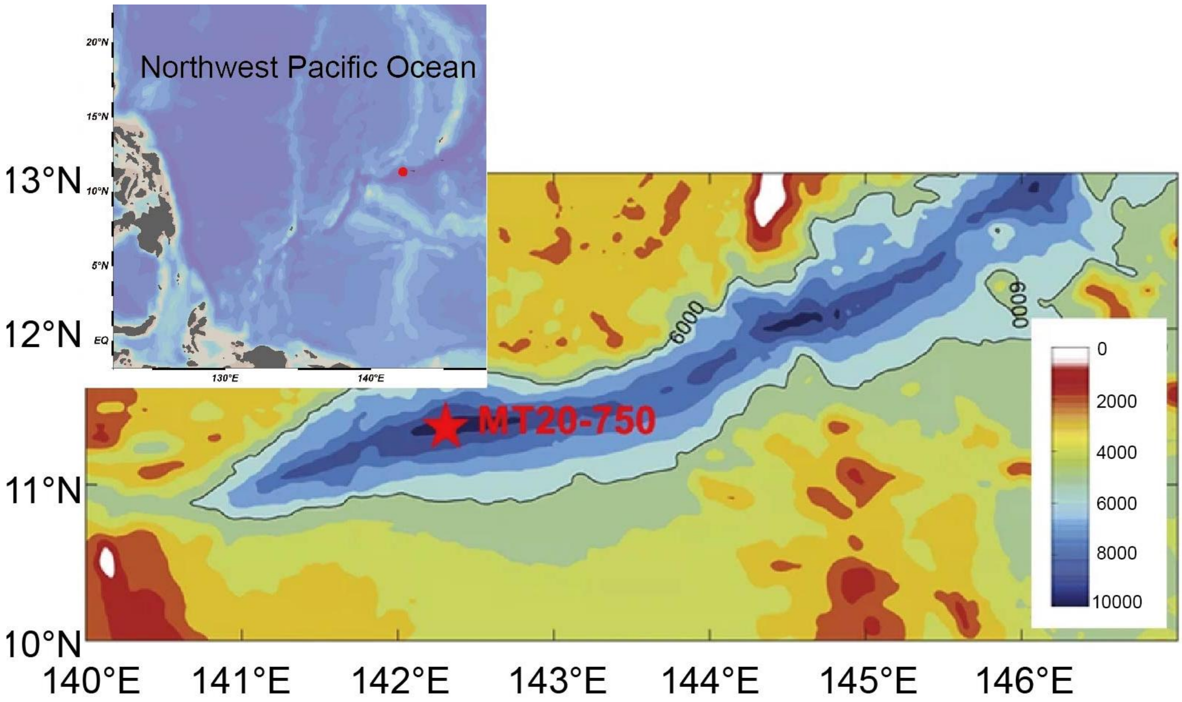

Fig. S11 Map showing ocean bathymetry of the southern Mariana Trench and the location of the core site MT20-750 (red star). The bathymetric data were sourced from the ETOPO1 (https://www.ngdc.noaa.gov/mgg/global/relief/ETOPO1/). Adapted with permission from [1].

References

1. Liu J, Li DW, He X, Liu R, Cheng H, Su C, Chen M, Wang Y, Zhao Z, Xu H, et al. A unique subseafloor microbiosphere in the Mariana Trench driven by episodic sedimentation. Mar Life Sci Technol. 2024;6:168-181. https://doi.org/10.1007/s42995-023-00212-y
